# Photosensitized direct C–H fluorination and trifluoromethylation in organic synthesis

**DOI:** 10.3762/bjoc.16.183

**Published:** 2020-09-03

**Authors:** Shahboz Yakubov, Joshua P Barham

**Affiliations:** 1 Fakultät für Chemie und Pharmazie, Universität Regensburg, Universitätsstraße 31, 93040 Regensburg, Germany

**Keywords:** C–H activation, energy transfer, fluorination, photocatalysis, photosensitization, visible light

## Abstract

The importance of fluorinated products in pharmaceutical and medicinal chemistry has necessitated the development of synthetic fluorination methods, of which direct C–H fluorination is among the most powerful. Despite the challenges and limitations associated with the direct fluorination of unactivated C–H bonds, appreciable advancements in manipulating the selectivity and reactivity have been made, especially via transition metal catalysis and photochemistry. Where transition metal catalysis provides one strategy for C–H bond activation, transition-metal-free photochemical C–H fluorination can provide a complementary selectivity via a radical mechanism that proceeds under milder conditions than thermal radical activation methods. One exciting development in C–F bond formation is the use of small-molecule photosensitizers, allowing the reactions i) to proceed under mild conditions, ii) to be user-friendly, iii) to be cost-effective and iv) to be more amenable to scalability than typical photoredox-catalyzed methods. In this review, we highlight photosensitized C–H fluorination as a recent strategy for the direct and remote activation of C–H (especially C(sp^3^)–H) bonds. To guide the readers, we present the developing mechanistic understandings of these reactions and exemplify concepts to assist the future planning of reactions.

## Review

### 1 Introduction

#### 1.1 Importance of direct C–H fluorination/trifluoromethylation and photosensitization in organic synthesis

**1.1.1 Importance of fluorine atoms in organic molecules:** Here, we briefly summarize the importance of fluorine atoms in organic molecules in the context of medicinal chemistry, materials chemistry, analytical chemistry and in the mechanistic studies of synthetic reactions. The importance of fluorination reactions in organic synthesis is reviewed exhaustively elsewhere, and the readers are referred to the relevant reviews [1–5].

**Figure 1:**
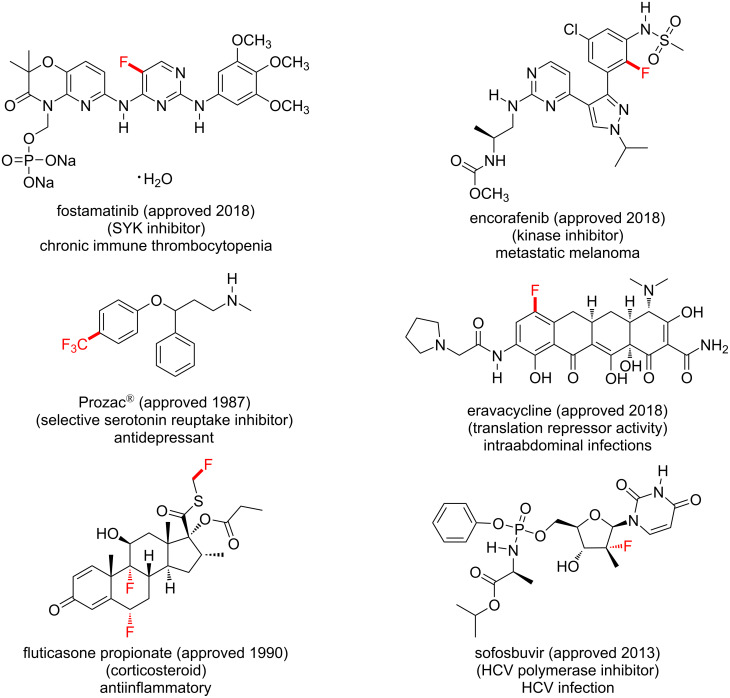
Fluorine-containing drugs.

**Figure 2:**
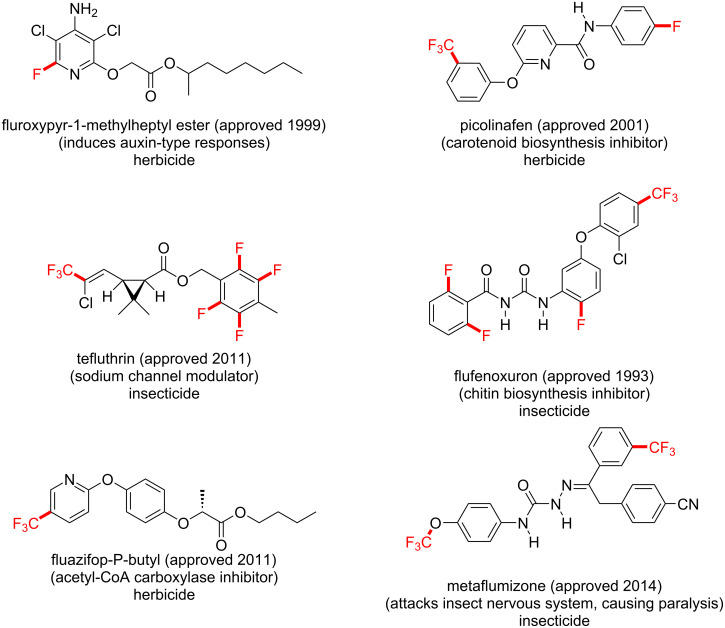
Fluorinated agrochemicals.

Over the last two decades, introducing fluorine atoms into molecules has become a crucial aspect in contemporary synthesis of pharmaceuticals. Extraordinarily, about half of the so-called “blockbuster drugs” contain fluorine atoms, including drugs used to treat cancer, HIV, smallpox and malarial infections (Figure 1) [5–6]. Moreover, fluorine atoms and trifluoromethyl groups have dramatic effects on the biological activity of agrochemicals, such as herbicides, insecticides and fungicides, as reflected by a plethora of fluorinated agrochemicals (Figure 2) [7–8]. Fluorine possesses some unique properties, such as the highest atomic electronegativity [9] and, in its molecular form (F_2_), the most positive standard reduction potential (*E*^0^) of +2.87 V [10], as predictable from its position in the periodic table. The C–F bond length is ≈1.35 Å and is one of the strongest bonds known (bond dissociation enthalpy (BDE) of ≈130 kcal⋅mol^−1^) [11–12]. By C(sp^3^)–H/C(sp^3^)–F substitution, adjacent C–C bonds are strengthened while allylic C–C double bonds are weakened [13]. Due to the similar size to the hydrogen atom (*r*_F_ = 1.47 Å, *r*_H_ = 1.20 Å) [9,14], the fluorine atom is often used as a bioisostere for the hydrogen atom. Furthermore, the trifluoromethyl group was recently identified as a bioisostere for the nitro (NO_2_) group, which is important due to the strong binding ability of the nitro group and the high reactivity, which is speculated to raise toxicity issues [15].

Despite their similar size to hydrogen atoms, fluorine atoms possess a very different chemical reactivity and exert very different influences on neighboring atoms. C–H/C–F substitution in organic molecules influences practically all physical, pharmacokinetic, metabolic stability, adsorption and excretion properties of the compound [16–17]. For example, the pharmacokinetics of drugs are mostly regulated by the balance of the lipophilicity, hydrophilicity and ionization state. Since C–H/C–F substitution in a drug molecule enhances the lipophilicity [18], this has a positive impact on the biological absorption and distribution. Moreover, C–H/C–F substitution strongly decreases the basicity of neighboring amines (and lowers the p*K*_a_ by a similar increment) due to the σ-inductive effect of F. This results in i) a higher oral bioavailability [19], ii) a potential liability for phospholipidosis [20], iii) an increased membrane permeability [21] and iv) the mitigation of undesired binding to the human ether a-go-go-related gene (hERG) K^+^ channel associated with cardiovascular toxicity [22]. Moreover, the radiochemical properties of the ^18^F nucleus render them crucial isotopes for positron emission tomography (PET) due to their suitable half-life (*t*_1/2_ = 110 min) and low positron energy, which allows the production of high-resolution images [23]. Therefore, radiopharmaceuticals that contain fluorine substituents are in high demand. Fluorine enhances important properties of polymers for their applications as water-repellent, chemical-proof, lubricant and thermally- and electrically-insulating materials [24]. The importance of fluorine atoms in chemical crop protection agents is well-acknowledged because fluorine improves the biological activity and user safety, decreases the environmental impact, and has new modes of action*.* In analytical chemistry, fluorine nuclei (^19^F) are ideal for quantitative integration in NMR spectroscopy, comparable to ^1^H NMR spectroscopy [25–26]. In contrast to ^13^C signals, fluorine signals are well-resolved, and the nuclear spin of fluorine (*I* = 1/2) allows the coupling with nearby carbon and hydrogen atoms to be resolved [27].

**1.1.2 Relevance of direct C–H fluorination methods:** Remarkable developments in synthetic chemistry over the last several decades have allowed the transformation of almost every organic functional group into another, paving the synthetic roadmap to many natural and unnatural compounds. While C–H bonds are not normally considered as functional groups, these are, however, ubiquitous, for example, in hydrocarbon fuels and in alkyl groups of countless organic molecules. The modification of unactivated C–H (especially C(sp^3^)–H) bonds into other functional groups would not only enormously expand the synthetic maneuverability but would also intensify the use of hydrocarbon feedstocks in organic synthesis. In this context, the advantage of direct C–H fluorination over traditional fluorination methods is that it does not require the introduction of other functional groups.

**Scheme 1:**
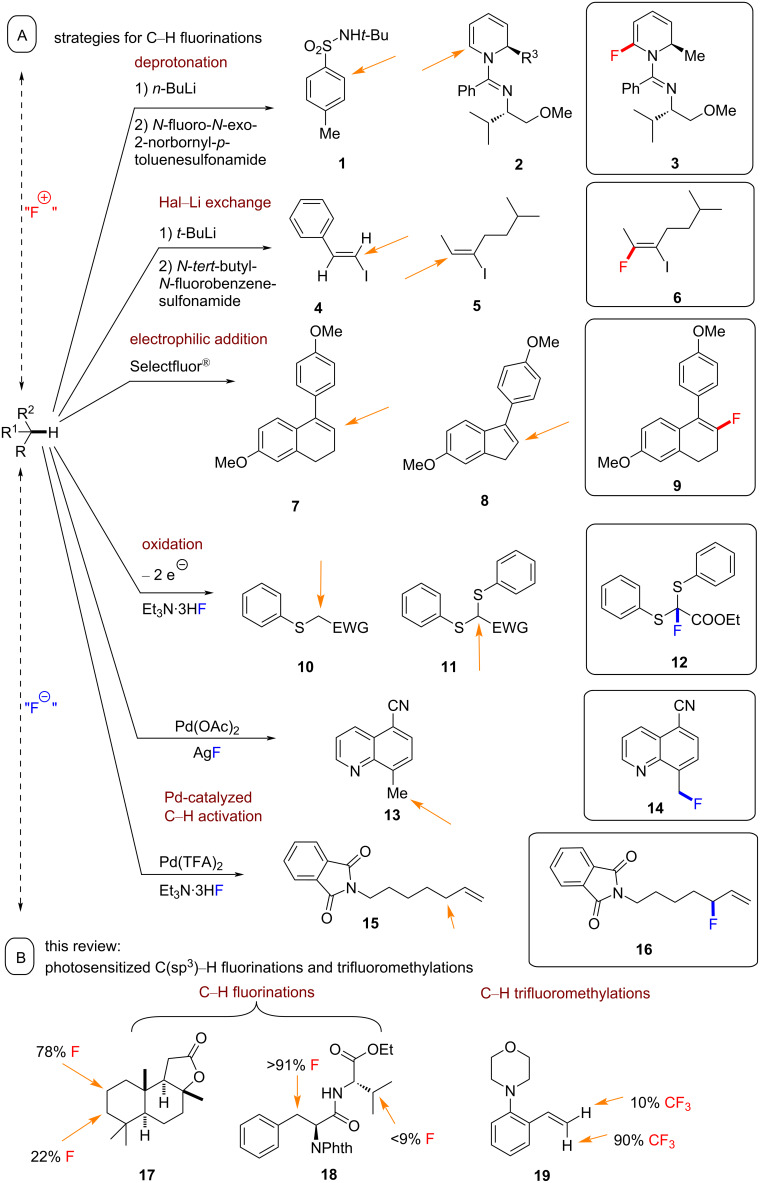
Selectivity of fluorination reactions.

Although many methods have been developed for fluorination reactions [3,28–46], including the addition of electrophilic fluorine to alkenes [1,28,32], the fluorination of aryl triflates [29] and arylpalladium complexes [2,47], few permit a one-step transformation of unactivated C(sp^3^)–H bonds to C(sp^3^)–F bonds [1,3,36,38,48]. Such a transformation would be highly valuable for the late-stage functionalization (LSF) of complex molecules, such as those in Scheme 1.

The lack of sophisticated methods for direct C(sp^3^)–H fluorinations is attributed to the challenges of regio- and chemoselectivity in C(sp^3^)–H activation. Compounds that possess several C(sp^3^)–H bonds will form several regioisomeric or polyfluorinated products that may be challenging to separate. Moreover, the inertness of C(sp^3^)–H bonds due to their high homolytic bond dissociation energy and low polarity necessitate highly reactive reagents/intermediates for their activation, which may encourage off-site activity with other functional groups present in the target molecule and lead to a poor chemoselectivity. Overall, chemo- and regioselective C(sp^3^)–H fluorinations continue to challenge chemists.

Most direct C(sp^3^)–H fluorinations are reported to proceed under radical pathways involving hydrogen atom transfer (HAT), although proton-coupled electron transfer (PCET) has also been reported [44,49–57]. Accordingly, the reactivity and selectivity correlate with the homolytic C–H bond dissociation enthalpy and polarity matching principles [58–64]. One of the advantages of polarity matching is that it enables C(sp^3^)–H bond activations that are not dictated by the thermodynamic driving force of the hydrogen atom transfer step (which depends on the stability of the resulting radical) or by the relative BDE of C–H bonds. Thus, it is possible to homolytically cleave stronger C–H bonds in the presence of weaker C–H bonds if the polarity of the stronger C–H bond is matched to the HAT catalyst. For example, an electrophilic radical abstracts H atoms selectively from the most electron-rich or “hydridic” C–H bond [65].

**1.1.3 Importance of visible light and visible-light photosensitization in synthesis:** Unlike many other traditional energy resources, light is inexpensive, nontoxic, noncontaminating, ample (or “limitless” in the case of sunlight) and a renewable source of energy for environmentally-friendly and “green” chemical synthesis. As a consequence of comprehending the detrimental impact of human industry on the environment, new methods to effectively utilize solar energy have represented a key scientific movement in the last two decades [66–68], especially after the oil crisis in the 1970s and the increasing solicitude about environmental pollution by chemical factories [69]. The attention on the solar generation of fuel brought unprecedented attention to photochemistry [70–71].

Increasing interest in photochemistry is often attributed to the ability to access radical intermediates that are otherwise difficult or impossible to obtain by classical chemistry but that can achieve unique chemical reactions complementary to two-electron processes [72–77]. However, the adoption of photochemistry into the toolbox of the synthetic organic chemist has been hindered by three phenomena: i) simple organic-molecule targets typically do not absorb (or have very small extinction coefficients for) visible light, which is among the most abundant in the solar distribution [78–79], instead absorbing only short wavelengths of UV light, ii) the photoexcitation of simple organic molecules by requisite high-energy UV photons populates higher-order excited states that undergo uncontrolled photodecomposition, such as Norrish-type cleavage reactions [80] and 1,5-HAT [81] and iii) the excited state of the simple organic molecule target can possess an ultrashort lifetime [82] that precludes photochemistry in favor of photophysical or nonradiative deactivation, e.g., fluorescence or internal conversion (IC).

**Scheme 2:**
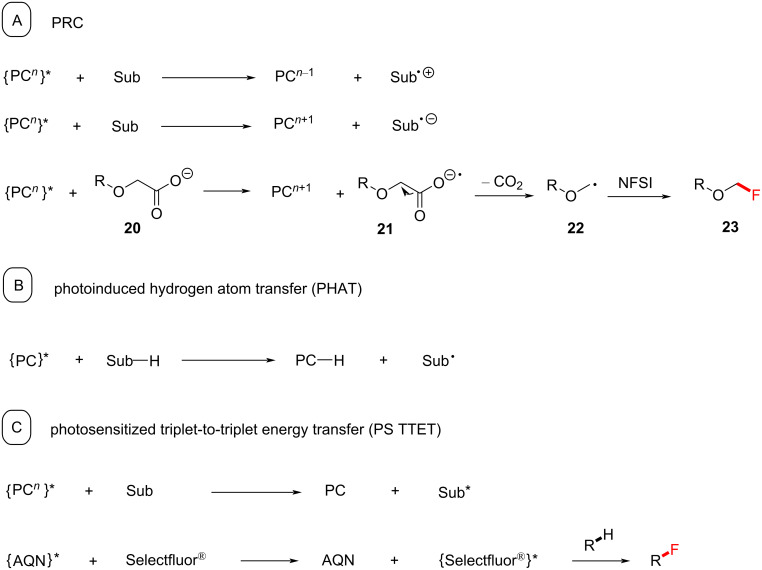
Different mechanisms of photocatalytic activation. Sub = substrate.

Instead of direct UV excitation of the target substrate, one solution is the use of visible-light-harvesting photocatalysts that transfer their excitation energy to the target substrate. Over the last few decades, the field of visible-light photocatalysis has proven itself as a highly effective, versatile and “green” strategy for inducing different organic transformations under extremely mild conditions without threatening reagents and conditions [83–85]. There are three commonly observed and distinct mechanisms of photocatalytic activation [86] in the context of organic synthesis:

i) Most reported photocatalytic reactions proceed under photoredox catalysis (PRC), involving Dexter electron transfer. Such photoredox reactions begin with the excitation of the photocatalyst (PC) by visible light, followed by a single-electron transfer (SET) between the excited photocatalyst and another molecule (quencher, Scheme 2A). An unfortunate consequence of this is that there are many organic molecules with redox potentials that lie beyond the range of those of the excited photocatalyst [87]. The transiently generated (ultralow concentration of) the excited-state catalyst does not persist long enough even for slightly endergonic SET, so no reaction occurs. Moreover, C–H bonds are not redox-active. To circumvent those issues, SET can be leveraged to generate an HAT agent to activate C–H bonds. For the purposes of this review, we label this reaction class involving redox processes as PRC.

ii) Photochemical reactions in which the excited state photocatalyst participates directly in HAT with the substrate (Scheme 2B), herein termed PHAT [88].

iii) Photochemical reactions where the photosensitization catalyst (PSCat) engages in Dexter energy transfer (typically TTET) with the substrate (or fluorinating reagent) to induce a chemical reaction (Scheme 2C)*.* This mechanistic class of PS TTET reactions is the focus of our review. PRC and PHAT processes are commonly grouped under the umbrella term “photocatalysis” in the literature, while PS TTET processes are often referred to as “energy transfer” [86].

**1.1.4 PRC vs photosensitized energy transfer:** Photocatalysis, photochemical sensitization and photosensitization are very similar terms. The IUPAC gold book deﬁnes photosensitization as follows: “The process by which a photochemical or photophysical alteration occurs in one molecular entity as a result of the initial absorption of radiation by another molecular entity called a photosensitizer. In mechanistic photochemistry, the term is limited to cases in which the photosensitizer is not consumed in the reaction” [89].

One can appreciate the close connection of this definition to that of photocatalysis, defined by the IUPAC as follows: “Change in the rate of a chemical reaction or its initiation under the action of ultraviolet, visible or infrared radiation in the presence of a substance – the photocatalyst – that absorbs light and is involved in the chemical transformation of the reaction partners” [90].

In principle, the photosensitizer is regenerated and therefore functions in a very similar way to a catalyst and can be deemed a photocatalyst. A common example of this concept is triplet photosensitization. The excited photocatalyst has a relatively high triplet-state energy and a long lifetime, such that it can function as an energy donor to activate the relatively lower triplet energy of the energy acceptor through an overall PS TTET process [91–100]. In contrast to PRC, the mechanism of TTET occurs in a way that does not depend on the redox potential of the molecules or on polar aprotic solvents, which are typically required for SET processes. Photosensitization also permits the facile and complementary access to radical intermediates via homolytic cleavage, without the need for SET [101–102].

**1.1.5 Justification and scope of the review:** Considering these advantages of photosensitization, it is clear that this field has attracted less attention than PRC methods. Moreover, PS TTET is a powerful method for the fluorination and trifluoromethylation of C–H bonds. We believe this may stem from the lack of dedicated reviews grouping together the theory [103–105], applications and mechanistic understanding that would enable researchers with a platform to plan photochemical reactions, as has been available for PRC [106–115]. Therefore, we present this review in the context of C–H fluorination and trifluoromethylation.

Section 2 equips readers with the basic theory of PS TTET and provides detailed property information tables on commonly utilized PSCats in organic synthesis and especially in C–H fluorinations, grouped by their properties relevant to PS TTET. Due to the importance of moving away from transition metal-based catalysts in organic synthesis and due to the exhaustive coverage of transition metal-based photosensitizers in the literature, we focus on detailing the properties of small-organic-molecule photosensitizers. Section 2 also highlights common fluorinating reagents, especially those used in C–H fluorinations. Section 3 groups the applications of direct C(sp^3^)–H fluorination by their achievement, either using PSCats or photosensitization auxiliaries (PSXs), while Section 4 presents the applications of trifluoromethylations involving photosensitization.

In order to guide the readers and to help with the planning of future reactions, throughout Sections 3 and 4, we present the developing mechanistic understanding of these reactions in the literature, including considerations such as triplet-to-triplet energy matching for photosensitization, polarity matching and directing groups for HAT. It is not our purpose to provide a detailed review on photosensitized energy transfer in organic synthesis, including a detailed account of the photophysical phenomena [105,116] nor is it our purpose to review C–H fluorinations accomplished by nonphotochemical methods or by photochemical methods deemed to proceed via SET.

### 2 Principles of photosensitization; selection of the photosensitizer and fluorinating reagent in C–H fluorinations

#### 2.1 Photosensitization and appropriate photosensitizer

**Figure 3:**
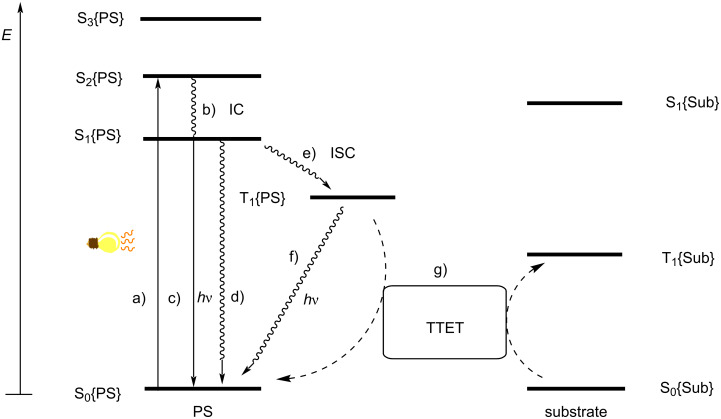
Jablonski diagram showing visible-light-induced energy transfer pathways: a) absorption, b) IC, c) fluorescence, d) radiationless transitions, e) ISC, f) phosphorescence and g) TTET.

The general mechanism of photosensitization is described in Figure 3 [86]. Initially, the PS absorbs visible light and is excited from the ground singlet state S_0_{PS} to the excited singlet state S_1_{PS}. Intersystem crossing (ISC) transforms S_1_{PS} to its more stable triplet state, T_1_{PS}. ISC is a process in which a nonradiative transition of a singlet excited electronic state to a triplet excited state takes place at a point where the potential energy curves of these two states interset, which requires spin–orbit coupling [117]. In the absence of spin–orbit coupling, it is a forbidden transition according to the Pauli exclusion principle. The resulting triplet state, T_1_{PS} is in an excited vibrational state that collides with nearby molecules to reach its lowest vibrational energy state.

**Figure 4:**
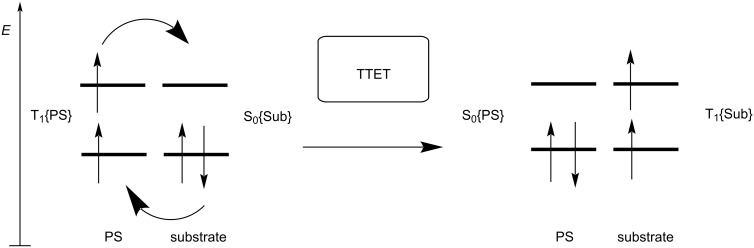
Schematic illustration of TTET.

Another fate of T_1_{PS}, permitted by spin–orbit coupling, is the radiative process of phosphorescence. If a molecule exhibits a high triplet quantum yield (rapid ISC and slow phosphorescence), this state is long-lived and can exist beyond the timeframe of diffusion, to be leveraged in photosensitization. Intermolecular energy transfer from T_1_{PS} to the substrate excites it from its ground singlet state S_0_{Sub} to its excited triplet state T_1_{Sub}. This intermolecular energy transfer process, known as TTET, involves the exchanges of two electrons with different spin and energy between two molecules (Figure 4). Therefore, it can be termed a Dexter energy transfer mechanism, not a Forster resonance energy transfer (FRET) mechanism. The generated triplet state of the substrate then undergoes further chemical transformation.

There are four prerequisites for TTET to be effective: i) The PS must have a chromophore that can absorb light, 2) the energy gap between S_1_{PS} and T_1_{PS} must be higher in energy than the energy gap between S_1_{Sub} and T_1_{Sub}, iii) ISC between S_1_{PS} and T_1_{PS} must be rapid to ensure a high quantum yield of the triplet state and iv) the energy transfer from the PS to the substrate must, overall, be exergonic. An ideal PS is a single, well-characterized pure substance of known composition that is stable at room temperature. In the presence of light, it is not toxic or degrades to toxic byproducts. It is inexpensive, sustainable (ideally not ruthenium or iridium complexes) and commercially available. Due to their comparatively high triplet energies and the long lifetime of their triplet states; organic dyes and various ruthenium and iridium complexes are well-suited photosensitizers [118–122].

**Figure 5:**
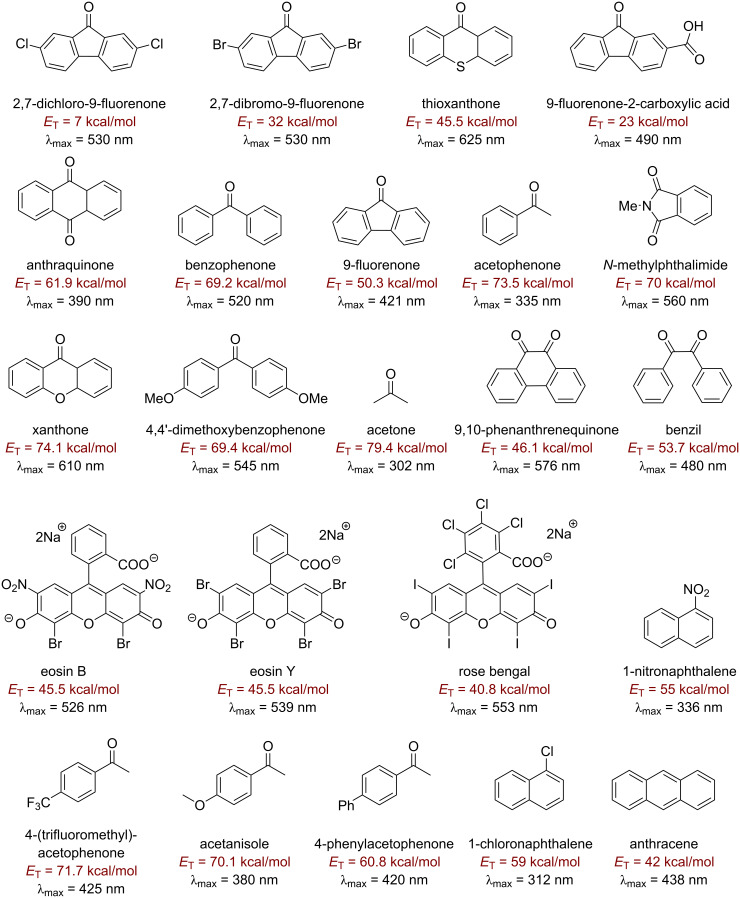
Organic triplet PSCats.

**Figure 6:**
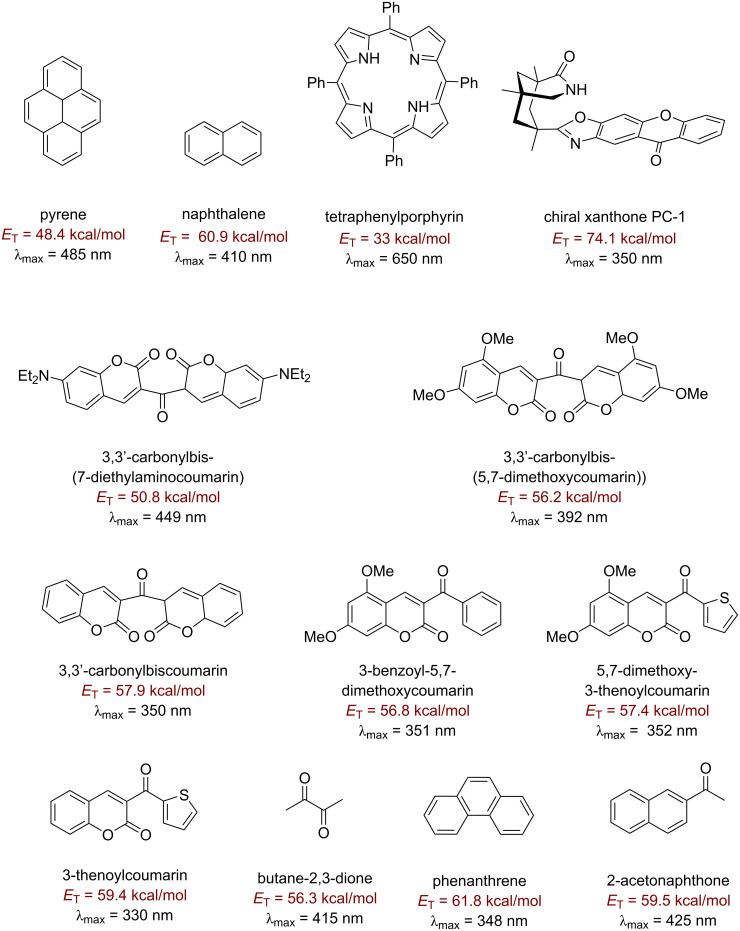
Additional organic triplet PSCats.

**Figure 7:**
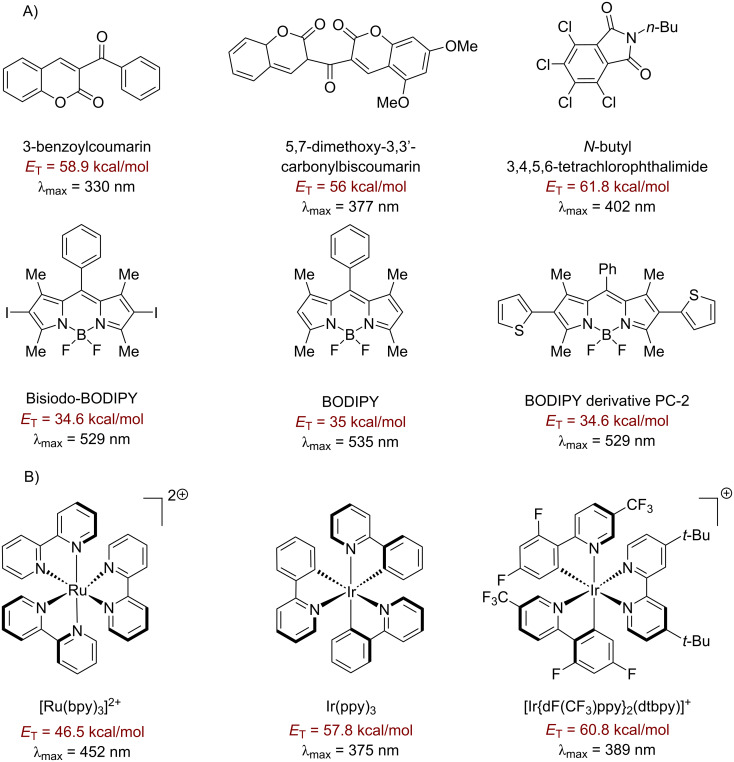
A) Further organic triplet PSCats and B) transition metal triplet PSCats.

There are many approaches to photosensitized fluorination that do not involve direct C–H activation, which are reviewed elsewhere [123–125], such as C–C bond fragmentation/C–F bond formation [126], aminofluorination of cyclopropanes [127] and decarboxylative fluorination [128]. These employ photosensitizers such as 9-fluorenone [127], 1,2,4,5-tetracyanobenzene (TCB) [129–130], 1,4-dicyanobenzene (CB) [126,131] and benzophenone [128] and proceed in low (23%) to excellent (96%) yields [126,128]. One of the most efficient and high-energy (79.4 kcal⋅mol^−1^) triplet photosensitizers is acetone. Frequently, it is used as a solvent or in high concentration where its optical density allows light absorption at approximately 300 nm, even though λ_max_ is about 270 nm [132]. Benzil, 9-fluorenone and 9,10-phenanthrenequinone are also well-established visible light triplet PSCats that are very cost-effective, commercially available and easy to handle [133–135]. Xanthone is capable of absorbing visible light (>400 nm) and has a triplet energy of about 74.1 kcal⋅mol^−1^ [136–139]. Figure 5 and Figure 6 as well as Table 1 show the structures and documented absorbance, triplet state energies and lifetimes of several small organic molecule/dye PSCats. Figure 7 shows the corresponding information for selected prototypical transition metal complexes.

**Table 1:** Absorption maxima, triplet energies and lifetimes of common PSCats.

Entry	PSCat	λ_max_ (nm)	*E*_τ_ (kcal⋅mol^−1^)	Lifetime of the triplet excited state (τ)	Reference

1	2,7-dibromo-9-fluorenone^a^	530	32.0	2.7 ns	[140–141]
2	2,7-dichloro-9-fluorenone^a^	530	7.0	8.5 ns	[141]
3	thioxanthone^a^	625	45.5	4.0 µs	[136,142]
4	9-fluorenone-2-carboxylic acid^a^	490	23.0	9.1 ns	[141]
5	anthraquinone^b^	390	61.9	0.2 µs	[137,143–144]
6	benzophenone^a^	520	69.2	0.7 µs	[136–137]
7	9-fluorenone^a^	421	50.3	16.5 ns	[136–137,141]
8	acetophenone^a^	335	73.5	0.2 µs	[136–137,145]
9	*N*-methylphthalimide^a^	350	70.0	5.0 µs	[146–148]
10	xanthone^b^	610	74.1	8.3 µs	[136–138,149]
11	4,4’-dimethoxybenzophenone^a^	545	69.4	–	[136–137]
12	acetone^a^	302	79.4	47.0 µs	[132,137,150]
13	9,10-phenanthrenequinone^a^	576	46.1	7.3 µs	[151]
14	benzil^a^	480	53.7	–	[137,152–153]
15	eosin B^c^	526	45.5	–	[154–155]
16	eosin Y^c^	539	45.5	–	[107,155–157]
17	rose bengal^d^	553	40.8	30.0 µs^a^	[107,137,158–159]
18	1-nitronaphthalene^e^	580	55.0	–	[137,160]
19	4-(trifluoromethyl)acetophenone^a^	425	71.7	–	[136–137]
20	acetanisole^a^	380	70.1	–	[136–137]
21	4-phenylacetophenone^a^	420	60.8	–	[136]
22	tetraphenylporphyrin^a^	650	33.0	–	[161]
23	1-chloronaphthalene^f^	312	59.0	–	[160,162]
24	anthracene^a^	438	42.0	–	[137,163]
25	pyrene^a^	485^g^	48.4	–	[160,164]
26	naphthalene^a^	410^g^	60.9	38 µs	[137,144,163–164]
27	chiral xanthone PC-1	350	74.1	–	[157]
28	3,3’-carbonylbis(7-diethylaminocoumarin)^a^	449	50.8	–	[165–166]
29	3,3’-carbonylbis(5,7-dimethoxycoumarin)^a^	392	56.2	–	[165–166]
30	5,7-dimethoxy-3,3’-carbonylbiscoumarin^a^	377	56.0	–	[165–166]
31	3,3’-carbonylbiscoumarin^a^	350	57.9	–	[165–166]
32	3-benzoyl-5,7-dimethoxycoumarin^a^	351	56.8	–	[165–166]
33	5,7-dimethoxy-3-thenoylcoumarin^a^	352	57.4	–	[165–166]
34	3-benzoylcoumarin^a^	330	58.9	–	[165–166]
35	3-thenoylcoumarin^a^	330	59.4	–	[165–166]
36	[Ru(bpy)_3_]^2+a^	452	46.5	1100–850 ns	[108,157,167]
37	Ir(ppy)_3_^a^	375	57.8	1.9 µs	[108,157]
38	[Ir{dF(CF_3_)ppy}_2_(dtbpy)]^+a^	389	60.8	2.3 µs	[108,157]
39	butane-2,3-dione	415	56.0	–	[160,168]
40	phenanthrene^a^	348	61.8	–	[160,169]
41	2-acetonaphthone^a^	425	59.5	–	[136]
42	bisiodo-BODIPY^a^	529	34.6^h^	57.1 µs	[170]
43	BODIPY^a^	503	35.0^h^	0.02 µs	[170]
44	BODIPY derivative PC-2^i^	529	34.6^h^	390 µs	[171]
45	*N*-butyl-4,5,6,7-tetrachlorophthalimide	402^j,k^	61.8^j,l^	–	[172]

The absorption maximum λ_max_ refers to the longest-shifted maximum towards, or in the visible region. ^a^In MeCN or MeCN (aq). ^b^In C_6_H_6_. ^c^In EtOH/MeOH 9:1. ^d^In DMF. ^e^EPA (77 K). ^f^In H_2_O. ^g^Chemisorbed onto alumina. ^h^Calculated *E*_T_. ^i^In PhMe. ^j^In cyclohexane. ^k^λ_max_ assumed from an *N-*substituted analog. ^l^Triplet energy assumed from phosphorescence.

#### 2.2 Fluorination reagents

An extensive discussion on the different types of fluorination reagents is beyond the scope of this review, and readers are directed to relevant reviews on the topic [8,173–174]. Here, we present a brief overview.

**Figure 8:**
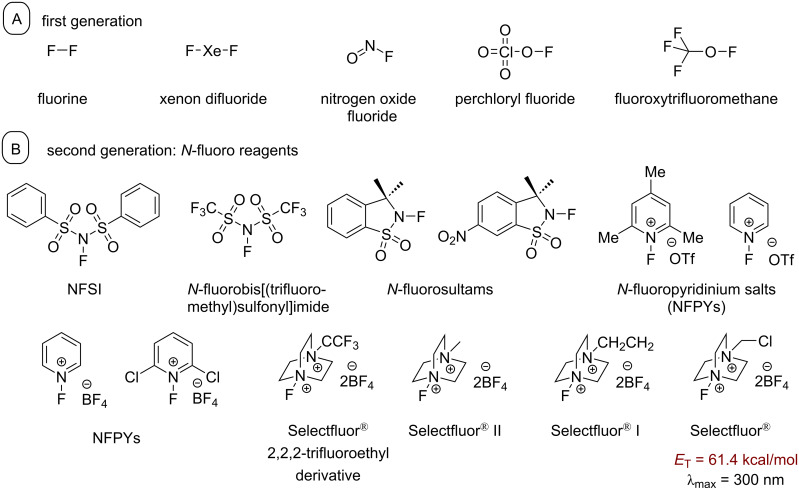
Different fluorination reagents grouped by generation.

Fluorine gas is the most fundamental electrophilic source of fluorine atoms; however, it is highly toxic, reactive and explosive, which severely limits its applicability in synthetic chemistry [175]. Despite the utility of (diethylamino)sulfur trifluoride (DAST) [176], morpholinosulfur trifluoride and tetrabutylammonium fluoride (TBAF) [4] as nucleophilic fluorine sources, the high reactivity of alternative electrophilic fluorine sources, such as fluoroxysulfates and hypofluorites [177–179], renders their employment in synthesis problematic. The high demand for a safe, stable and highly reactive electrophilic fluorinating reagent prompted researchers to synthesize the first generation of electrophilic fluorination reagents, including fluoroxytrifluoromethane [180], fluorine perchlorate [181], xenon difluoride [182], nitrogen oxide fluorides [183] and several other hypofluorides [184–185] (Figure 8A).

The second generation of fluorine reagents were the *N*-fluoro reagents (Figure 8B). These possessed selectivity, functional group tolerance, were safer and less expensive. Umemoto and co-workers first synthesized the NFPYs [186]. Further synthesis of similar compounds resulted in the synthesis of *N*-fluorobis(phenyl)sulfonimide (NFSI) [187], *N*-fluorobis[(trifluoromethyl)sulfonyl]imide [188–189], *N*-fluoropyridinium salts [186], *N*-fluorosaccharinsultam and 4-nitro-substituted *N*-fluorosaccharinsultam. The most recent discovery of an advanced electrophilic fluorinating agent was Selectfluor^®^ (1-chloromethyl-4-fluoro-1,4-diazoniabicyclo[2.2.2]octane bis(tetrafluoroborate)) [190].

**Scheme 3:**
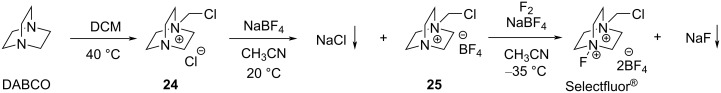
Synthesis of Selectfluor^®^.

Selectfluor^®^ [190–191] is a nonhygroscopic, crystalline solid, stable at temperatures up to 195 °C, nonhazardous [192], reliable and commercially available [193]. Selectfluor^®^ is synthesized on a multiton p.a. scale in a simple and efficient method (Scheme 3) [193]. The precursor **24** is prepared by alkylation of DABCO (1,4-diazabicyclo[2.2.2]octane) with DCM. A counterion exchange with NaBF_4_ causes NaCl precipitation in MeCN, and the fluorination with F_2_ in the presence of sodium tetrafluoroborate affords Selectfluor^®^. Moreover, by variation of these conditions, different derivatives of Selectfluor^®^ (e.g., a methyl derivative: Selectfluor^®^ II and a 2,2,2-trifluoroethyl derivative) with different physical properties and reactivity can be synthesized on an industrial scale (Scheme 3) [194].

#### 2.3 General mechanism of photosensitized C–H fluorination

Generally, the mechanism of photocatalytic activation induced by energy transfer involves the simultaneous photoinduced electron exchange between the photosensitizer and the substrate. It is necessary to note that this process does not imply any net redox chemistry. For this reason, it is difficult to predict the reactivity of these processes by the electrochemical potentials of the compounds. On the contrary, the triplet state energy of the photocatalyst and the reactant are very useful to determine the possibility of an energy transfer process.

**Scheme 4:**
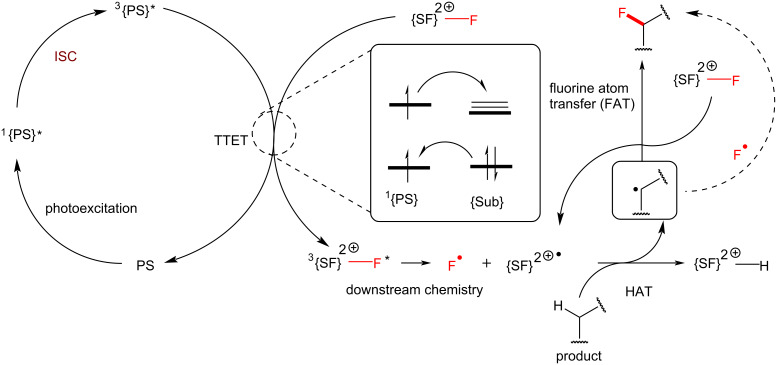
General mechanism of PS TTET C(sp^3^)–H fluorination.

At the first stage of the mechanism, the photocatalyst is excited from the singlet ground state to the excited singlet state (Scheme 4) [86]. Ordinarily, most organic molecules do not undergo effective ISC to the triplet state, consequently they relax quickly to the ground state. However, triplet photosensitizers undergo rapid ISC and successfully generate long-lived triplet states. After an energy transfer between the triplet excited photosensitizer and the substrate (e.g., Selectfluor^®^), the photocatalyst is relaxed to the ground state and the substrate is excited to its triplet excited state to undergo downstream chemistry. In the case of triplet Selectfluor^®^, plausible mechanisms for the downstream chemistry are shown in Scheme 4 and discussed in more detail in the following section.

### 3 C–H fluorination of visible-light-inactive molecules using PSCats

#### 3.1 Benzylic C–H fluorination

**Scheme 5:**
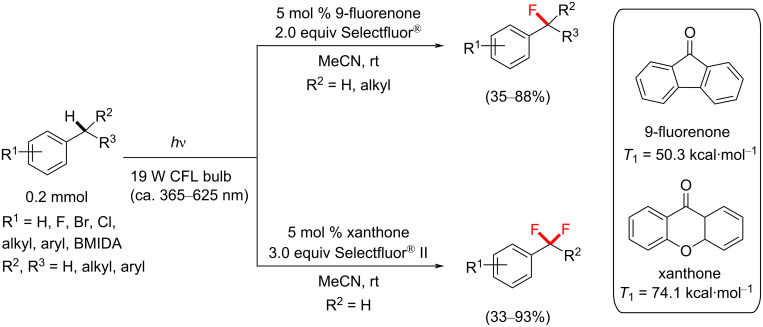
Selective benzylic mono- and difluorination using 9-fluorenone and xanthone PSCats, respectively.

A seminal paper in the field of transition-metal-free direct C–H fluorination comes from Chen and co-workers, who applied aryl ketones as PSCats [135]. They discovered that the choice of the PS impacted the selective formation of mono- vs difluorinated products at the benzylic position; 9-fluorenone affords benzylic monofluorination and xanthone affords benzylic difluorination (Scheme 5). Importantly, this metal-free direct C–H fluorination proceeded without the need for any specialized photochemical equipment; under visible-light irradiation by a household (19 W) compact fluorescent light (CFL) bulb (emitting variable wavelengths in the range of ≈365–625 nm) [195] and under mild conditions. Control reactions revealed that both the PS and light were essential for the reaction to occur. According to their report, benzylic C−H monoﬂuorination photosensitized by 9-fluorenone demonstrated a remarkable scope and reaction eﬃciency.

**Scheme 6:**
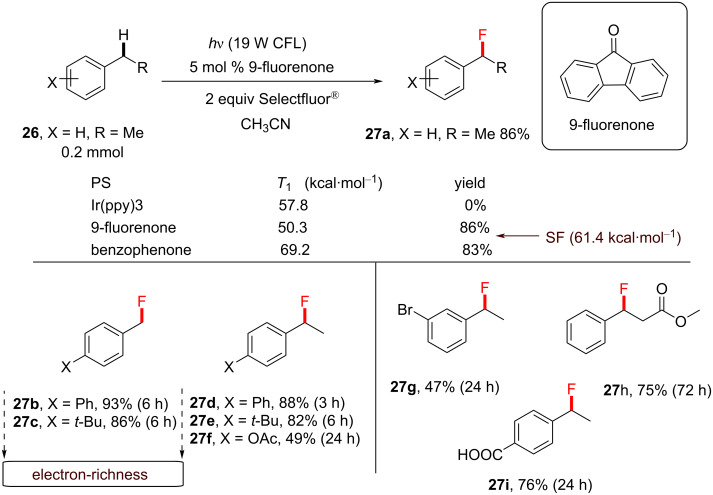
Chen’s photosensitized monofluorination: reaction scope.

A range of different photosensitizers (acetone, benzophenone, 9-fluorenone and Ir(ppy)_3_) were investigated. Acetone as a PSCat gave no product, likely due to its absorption bands (≈280 nm) well out of reach of even the lowest emission wavelength range of the CFL bulb. Ir(ppy)_3_ also gave no reaction. This was interesting, considering that its triplet energy (*T*_1_ = 57.8 kcal⋅mol^−1^) was similar to Selectfluor^®^ (*T*_1_ = 61.4 kcal⋅mol^−1^). Benzophenone (*T*_1_ = 69.1 kcal⋅mol^−1^) and 9-fluorenone (*T*_1_ = 50.3 kcal⋅mol^−1^) were most effective, affording 83% and 86% yield of the monofluorinated product **27a**, respectively (Scheme 6). A range of electrophilic fluorine sources was evaluated (for example, NFSI and *N-*fluoropyridinium salts), but Selectfluor^®^ was chosen for giving the highest yield of **27a**. Electroneutral and electron-poor compounds were successfully monofluorinated in modest to excellent (36–93%) yield of **27b**–**i** (Scheme 6). The functional group tolerance included bromoarenes, esters and unprotected benzoic acids.

**Scheme 7:**
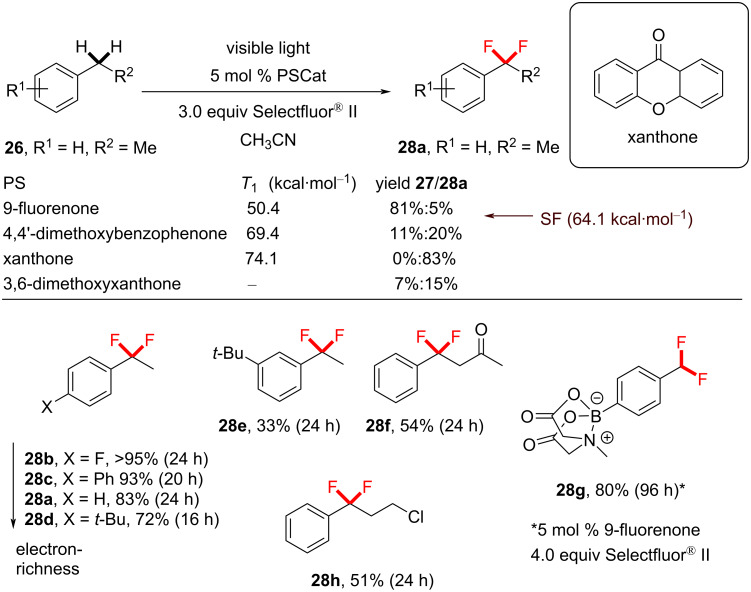
Chen’s photosensitized benzylic difluorination reaction scope.

Chen’s photosensitized approach represents the first photochemical C−H *gem*-diﬂuorination. *gem*-Difluorination is particularly challenging since the first introduced fluorine atom will electronically hinder the second fluorination at the same position. For difluorination, xanthone was employed as the PC and Selectfluor^®^ II as the fluorine source (Scheme 7). The higher *T*_1_ energy may have been responsible for generating more Selectfluor^®^-derived radical cations that drove the reaction towards difluorination to afford the product **28a**. However, it is important to note that only difluorinated products were detected and no monofluorinated products were observed, regardless of whether Selectfluor^®^ or Selectfluor^®^ II was employed. Were mono- and difluorination to proceed via the same mechanism, one would expect to see traces of the monofluorinated product **27a** unless the reaction of **27a** was faster than the reaction of the starting material **26**. Interestingly, NFSI gave exclusive monofluorination, albeit in a low (26%) yield, despite its lower BDE (calculated BDE = 63.4 kcal⋅mol^−1^) than Selectfluor^®^ [196]. *gem*-Difluorinations using xanthone gave a higher conversion of **26** and a higher yield of **28a** with Selectfluor^®^ II rather than with Selectfluor^®^ (Scheme 7), despite their identical N–F bond dissociation enthalpies (calculated BDEs = 64.0 kcal⋅mol^−1^) [196].

While the *T*_1_ energy of Selectfluor^®^ II is not reported to our knowledge, it is possible that the greater reactivity towards *gem*-difluorination could stem from it having a *T*_1_ energy closer to xanthone. The reaction conditions tolerated various electron-rich and electron-poor substituents, affording exclusively benzylic C(sp^3^)–H *gem*-difluorinated products in modest to excellent (33–95%) yield of **28b**–**h**. The functional group tolerance was demonstrated by successful reactions of alkyl chlorides, ketones and a *para*-substituted MIDA ester. A common trend between mono- and difluorination was that increasing the electron-releasing ability of a substituent *para* to the benzylic position decreased the product yield. It is known that polarity matching effects are negligible for benzylic HAT and that BDEs do not correlate with Hammett parameters of *para* substituents [197], and therefore the reaction success could relate to the transition state of the benzylic radical’s fluorination step.

**Scheme 8:**
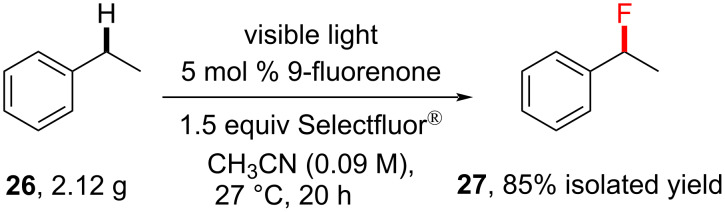
Photosensitized monofluorination of ethylbenzene on a gram scale.

Given later studies [147,198–199], it cannot be ruled out that this reaction could be interpreted as a photosensitized C–H fluorination involving the complexation of the PSCat with Selectfluor^®^ and TTET. The practicality of this photosensitized monoﬂuorination was demonstrated using ethylbenzene on gram scale (Scheme 8) [135]. Ethylbenzene (2.12 g) was monofluorinated to give the desired fluorinated product (2.11 g) in 85% isolated yield, almost identical to the 83% yield under the standard conditions. This reaction was also demonstrated in continuous flow under similar conditions [139].

#### 3.2 Polarity matching-guided unactivated C(sp^3^)–H fluorination

**Scheme 9:**
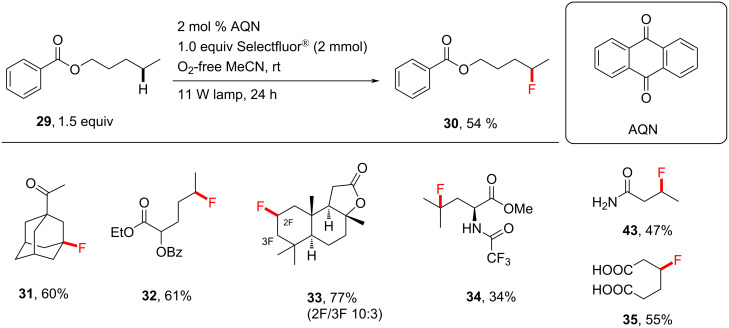
Substrate scope of Tan’s AQN-photosensitized C(sp^3^)–H fluorination.

**Scheme 10:**
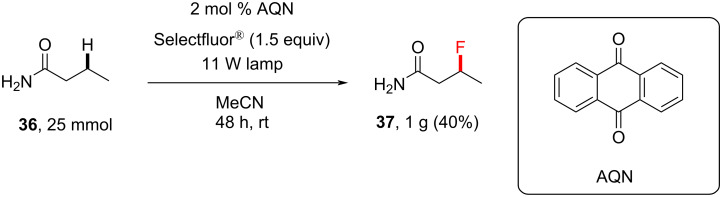
AQN-photosensitized C–H fluorination reaction on a gram scale.

**3.2.1 Synthetic applications:** In another seminal paper, Tan and co-workers [198] discovered the photocatalytic direct fluorination of unactivated C(sp^3^)–H bonds by employing Selectfluor^®^ and anthraquinone (AQN, *T*_1_ = 61.9 kcal⋅mol^−1^) as a photosensitizer. Control experiments showed that, under their conditions, both light and AQN were necessary for the reaction to proceed. A variety of different compounds containing multiple C(sp^3^)–H bonds and different functional groups were successfully fluorinated (Scheme 9). Free amide NH_2_ groups were tolerated with good yield (as for **43**). Although free amine groups were not tolerated, their protection with a trifluoroacetyl group enabled monofluorination, for example, to give **34** (34%). The monofluorination of butyramide on a 25 mmol scale successfully afforded 1.0 g of **37** in 40% yield, compared to 47% on a 3.0 mmol scale (Scheme 10).

Tan and co-workers noticed the selectivity difference in Chen’s previous report when NFSI and Selectfluor^®^ were employed [198], which led them to propose that Selectfluor^®^ is not only a fluorine source but that its radical cation participates as a HAT agent. When they substituted AQN (*T*_1_ = 61.9 kcal⋅mol^−1^) for 9-fluorenone (*T*_1_ = 50.3 kcal⋅mol^−1^) or alizarin red S (*T*_1_ = 34.0 kcal⋅mol^−1^), insignificant amounts of the fluorinated amyl benzoate **38** were formed. Since the triplet energy of AQN (2.7 eV) [200] is greater than/matched to the singlet–triplet energy gap of Selectfluor^®^ (2.66 eV) but the triplet energy of 9-fluorenone (2.36 eV) [137] and alizarin red S are not, they proposed, for the first time, that TTET between AQN and Selectfluor^®^ was the operating mechanism.

**Scheme 11:**
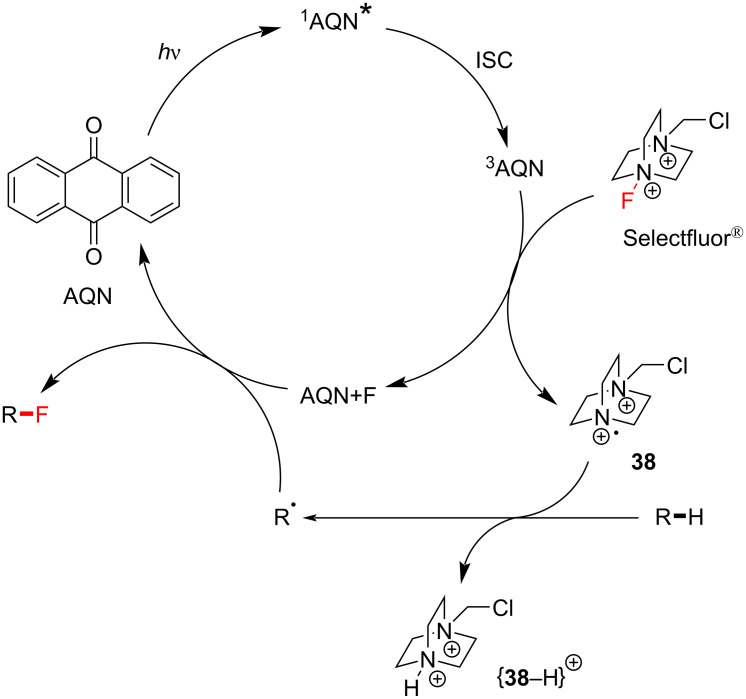
Reaction mechanism of the AQN-assisted fluorination.

**Figure 9:**
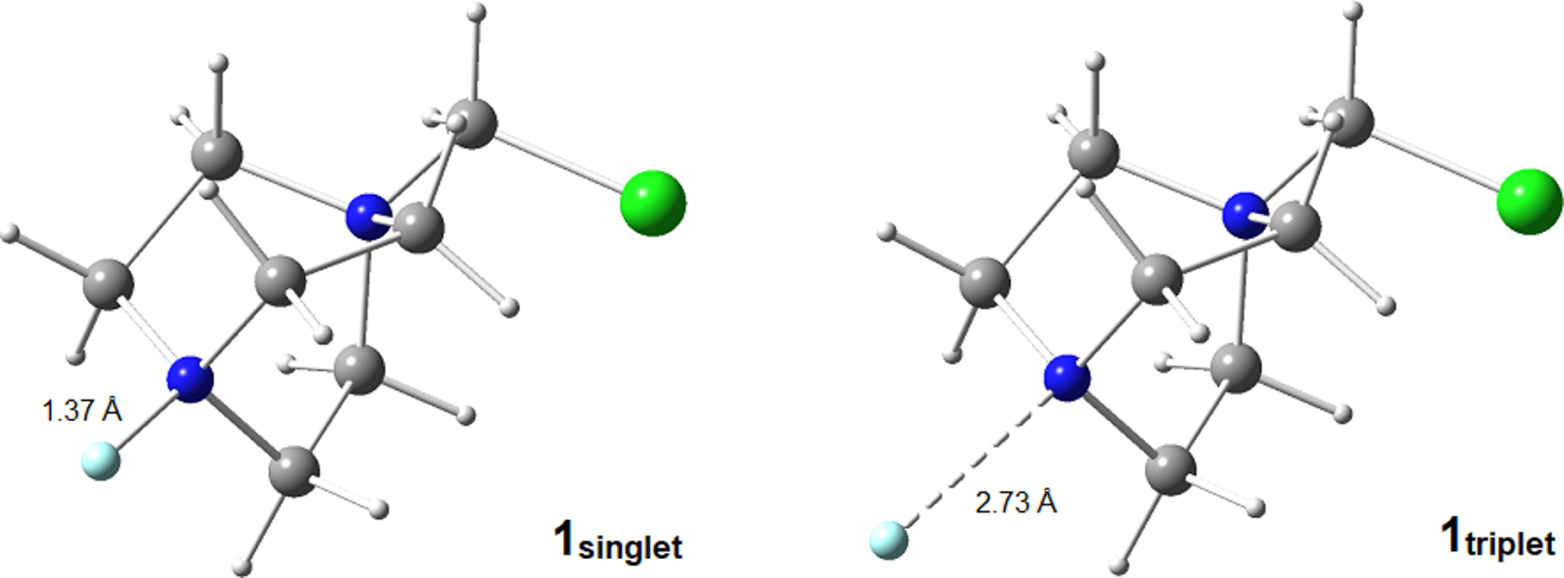
3D structures of the singlet ground and triplet excited states of Selectfluor^®^.

According to Tan’s proposed mechanism at that time (Scheme 11), AQN is photoexcited by visible light and triplet ^3^AQN* is generated upon ISC. TTET occurs between ^3^AQN* and Selectfluor^®^ to afford triplet ^3^[Selectfluor^®^]* that has a notably longer N–F bond (2.73 Å) compared to singlet Selectfluor^®^ (N–F = 1.37 Å, Figure 9). After the immediate dissociation of triplet Selectfluor^®^, the formed Selectfluor^®^ *N-*radical cation undergoes HAT with the substrate to afford an alkyl radical. The authors deemed a complex between AQN and fluorine (AQN + F) more plausible than the formation of fluorine radicals. The generated alkyl radical could abstract fluorine atoms either from i) the AQN–F complex to regenerate AQN or ii) Selectfluor^®^ to regenerate the Selectfluor® radical cation and thereby propagate a chain reaction.

**Scheme 12:**
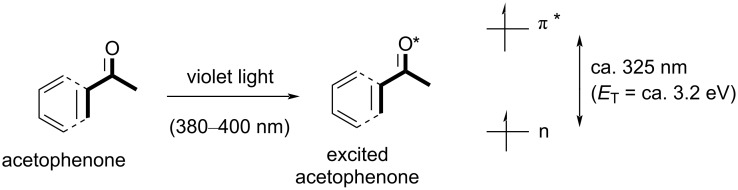
Associated transitions for the activation of acetophenone by violet light.

**Scheme 13:**
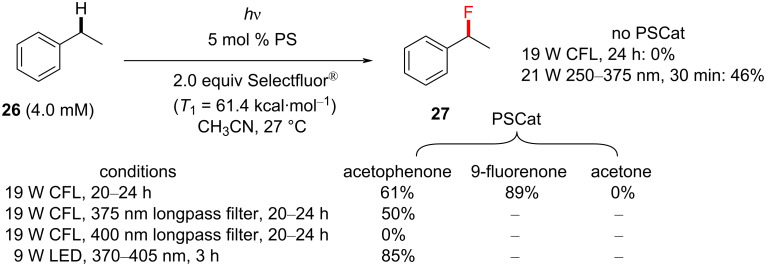
Ethylbenzene C–H fluorination with various PSCats and conditions.

Following shortly after Tan’s report, Chen and co-workers disclosed the direct C–H fluorination [201] of unactivated alkyl C(sp^3^)–H bonds using acetophenone as a PSCat, which was photoexcited by near-UV light (375–400 nm, Scheme 12). In the absence of a PSCat and using dedicated UV-light irradiation (275–375 nm), the product was observed together with numerous decomposition products (Scheme 13). No reaction was observed using a 19 W CFL bulb in the absence of a PSCat. Despite acetophenone being a colorless oil with only trace absorption above 375 nm (an n–π* transition corresponding to a photon of 325 nm), it successfully functioned as a PSCat, undergoing photoexcitation by a CFL to afford the C(sp^3^)–H fluorinated product in 85% yield (Scheme 13). Through the use of longpass filters, the authors found that wavelengths between 370–400 nm were necessary for the reaction to proceed.

**Scheme 14:**
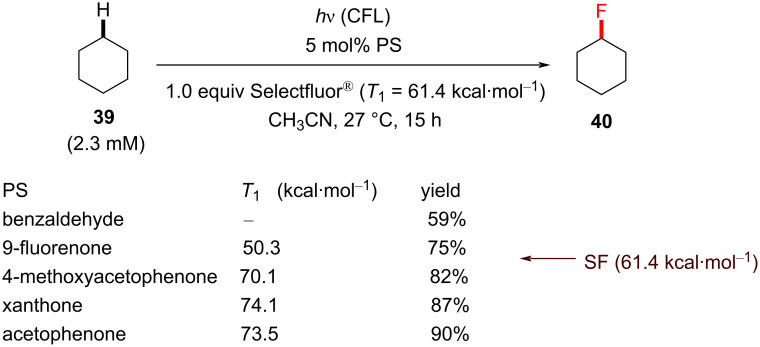
Effect of different PSCats on the C(sp^3^)–H fluorination of cyclohexane (**39**).

**Scheme 15:**
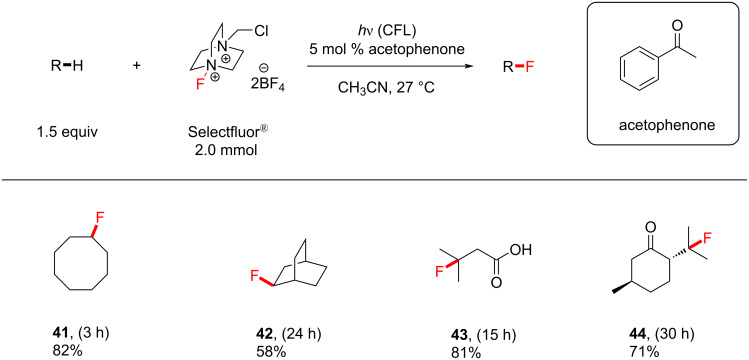
Reaction scope of Chen’s acetophenone-photosensitized C(sp^3^)–H fluorination reaction.

The authors then subjected cyclohexane (**39**) to similar conditions to examine the choice of the PSCat. Acetophenone derivatives as well as benzaldehyde, 9-fluorenone, benzophenone derivatives and xanthone were all examined with respect to catalytic loadings (5 mol %), and acetophenone remained the most effective. Despite having stronger absorption bands than acetophenone at visible wavelengths (>400 nm), 9-fluorenone and benzophenone were in fact less effective. The authors rationalized the different efficacies of the PSCats by their different n–π* energy gaps, of which acetophenone was among the largest and gave the highest yield of **40**. Scheme 14 shows the influence of *T*_1_ on the increasing yield of **40**. It was found that decreasing the substrate loading below 1.5 equiv was detrimental to the yield, so 1.5 equiv of the substrate was chosen as optimal. A variety of unactivated C(sp^3^)–H-containing substrates were monofluorinated in good to excellent yields (55–85%), obtaining the products **41**–**44** (Scheme 15).

Chen and co-workers maintained that their reaction proceeded via HAT between the excited-state ketone and the C(sp^3^)–H-containing substrate. However, a PS TTET mechanism still could not be ruled out when Selectfluor^®^ is present. That 9-fluorenone gave no reaction in Tan’s initial report [198] indeed presents a challenge to rationalizing Chen’s fluorinations of benzylic or unactivated C(sp^3^)–H bonds as PS TTET processes, giving credibility to a photoexcited HAT process. Herein, we note the convincing trend between the *T*_1_ energy and the reaction yield in their screening of PSCats (Scheme 14). However, the differences in the light sources and a radical-chain mechanism [202], which is initiated by PS TTET, cannot be conclusively ruled out.

**Figure 10:**
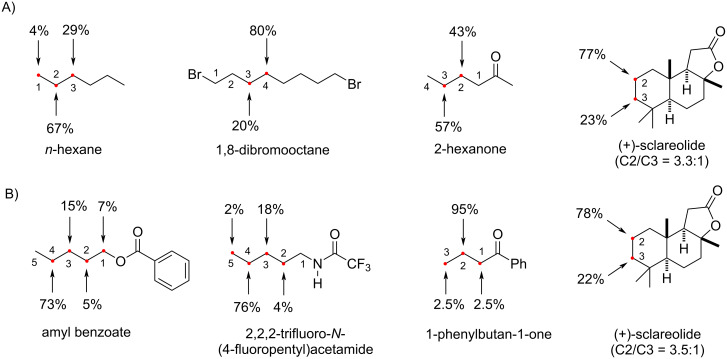
a) Site-selectivity of Chen’s acetophenone-photosensitized C–H fluorination reaction [201]. b) Site-selectivity of Tan’s AQN-photosensitized C–H fluorination reaction [198].

**3.2.2 C(sp^3^)–H fluorination selectivity:** Another facet that suggests a similar general mechanism is the C(sp^3^)–H fluorination selectivity. One might expect excited triplet states to be much more reactive and less selective than the Selectfluor^®^ radical cation. The selectivity in both Chen’s and Tan’s report is depicted in Figure 10. In both reaction conditions, employing either acetophenone or AQN as PSCat, the most electron-rich, or ”hydridic” C(sp^3^)–H bond that forms a secondary radical is predominantly fluorinated. This indicates that these reactions operate by HAT, which is directed by polarity matching effects [58–60]. For example, *n*-hexane is mainly fluorinated at its C2 position (67%) and only 4% fluorination at C1 occurs [201], due to the instability of the primary radical (Figure 10). The selective fluorination of C2 over C3 may be rationalized by steric effects (rather than polarity matching), which are reported for quinuclidinium radical cations [203].

A similar selectivity for the most hydridic C(sp^3^)–H bond was observed for amyl benzoate, a trifluoroacetylated pentylamine [198] and 1,8-dibromooctane [201]. 2-Hexanone was fluorinated with a slightly higher selectivity for C3 over C2 fluorination [201]. Interestingly, 1-phenylbutan-1-one gave nearly exclusive C2 fluorination [198]. Both fluorination positions of (+)-sclareolide (C2 and C3) are remote from the lactone EWG and both form secondary radicals. Thus, the fluorination of these positions is presumably dictated by hindrance, given that the C3 position is neighbored by a *gem*-dimethyl group and is fluorinated to a lesser extent (22%). In both reports, the C2/C3 selectivity for the photochemical monofluorination of (+)-sclareolide was identical, suggesting a similar mechanism behind the HAT and FAT processes. This parallels the C2/C3 selectivity for the electrochemical fluorination of (+)-sclareolide, proposed to involve a Selectfluor^®^ radical cation [204].

**Scheme 16:**
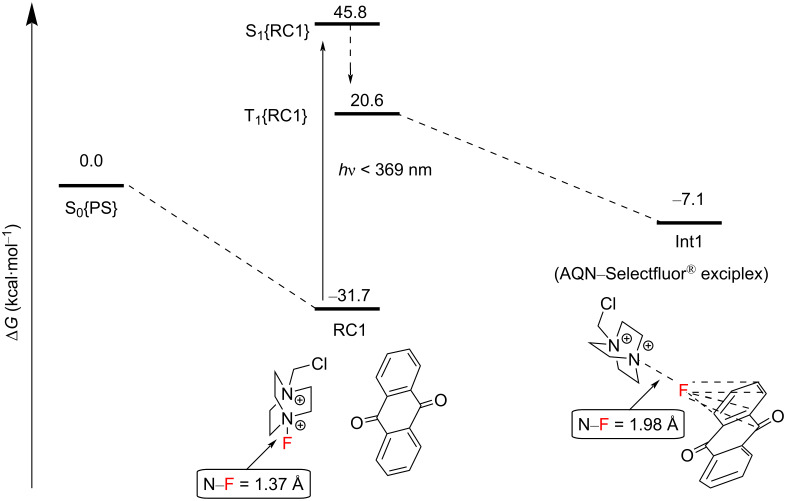
Formation of the AQN–Selectfluor^®^ exciplex Int1.

**3.2.3 C(sp^3^)–H fluorination mechanistic studies:** In order to unearth a greater understanding of their previously-reported photosensitized C(sp^3^)–H fluorination, Tan, Lu, Soo and co-workers employed a nanosecond-scale transient absorption (TA) spectroscopy and time-dependent density functional theory (TDDFT) calculations [199]. They were able to experimentally demonstrate, for the first time, that the reaction between photoexcited AQN and Selectfluor^®^ afforded a transient AQN–Selectfluor^®^ triplet exciplex species by calculating the predicted TDDFT absorption spectrum that matched with the experimentally obtained TA spectra. The direct reaction between AQN and the substrate was not observed by TAS. The authors’ DFT calculations revealed reaction pathways that were thermodynamically and kinetically plausible. Initially, AQN and Selectfluor^®^ (S_0_ in Scheme 16) form a van der Waals complex RC1, which is markedly more stable than its two individual components. Instead of the photoexcitation of AQN, photoexcitation of RC1 by a 369 nm photon (77.5 kcal⋅mol^−1^) affords the short-lived excited singlet state of RC1, S_1_ (Scheme 16). Nonradiative ISC occurs from S_1_ to yield the excited triplet state of RC1 (*T*_1_), and a spontaneous intramolecular reorganization process affords the AQN–Selectfluor^®^ exciplex Int1.

**Scheme 17:**
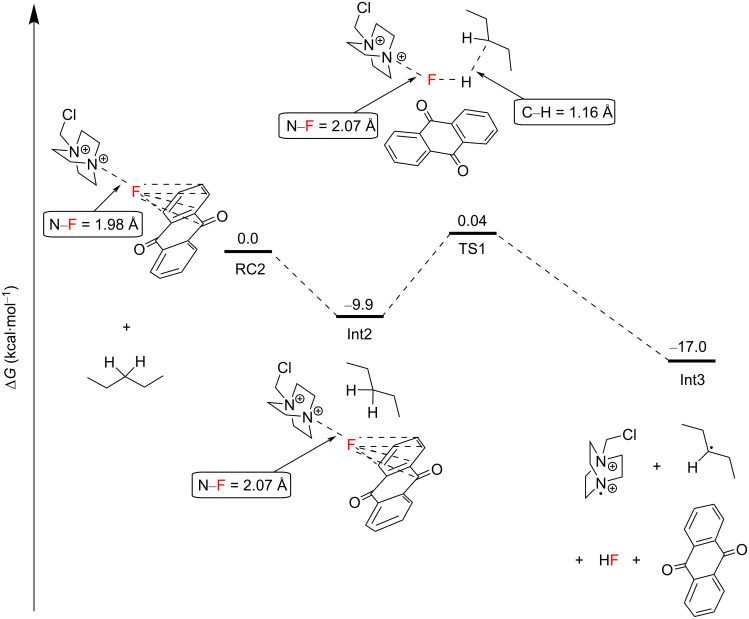
Generation of the C3 2° pentane radical and the Selectfluor^®^ *N-*radical cation from the exciplex.

The AQN–Selectfluor^®^ exciplex Int 1 possesses a longer N–F bond distance (1.98 Å) compared to the N–F bond distance (1.37 Å) of Selectfluor^®^ (Scheme 17). This indicates that the fluorine atom in the exciplex is stabilized by the aromatic system of AQN and that the N–F bond becomes more labile. In this exciplex, the spin density is delocalized mostly over the AQN–F moiety (61%), and the absorption band depends mainly on the AQN–F interactions. Thus, the authors concluded that the TA spectra should not depend on the electrophilic fluorinating source. Thereafter, the AQN–Selectfluor^®^ exciplex abstracts a hydrogen atom from the C3 position of pentane to form a secondary radical, a Selectfluor^®^ *N-*radical cation, HF and AQN. The activation energy barrier relative to RC2 was only 9.9 kcal⋅mol^−1^ (Scheme 17).

**Scheme 18:**
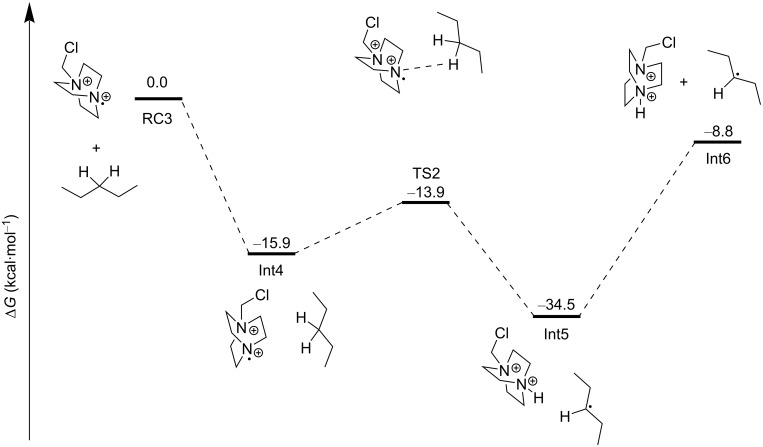
Hydrogen atom abstraction by the Selectfluor^®^ *N-*radical cation from pentane to give the C3 2° pentane radical.

**Scheme 19:**
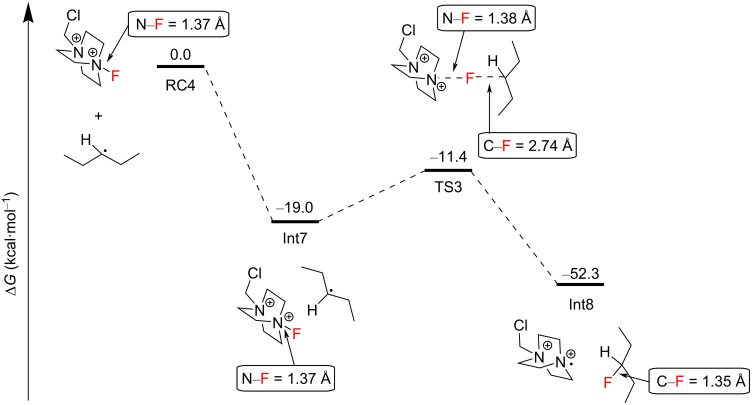
Fluorine atom transfer from Selectfluor^®^ to the C3 2° pentane radical to yield 3-fluoropentane and the Selectfluor^®^ *N-*radical cation.

The Selectfluor^®^ *N-*radical cation can abstract a hydrogen atom from the C3 2° C–H bond of another pentane molecule in an overall exergonic process with an activation energy barrier of 2.0 kcal⋅mol^−1^ from Int4 to afford Int5 and ultimately Int6 (Scheme 18). The authors did not mention the possibility of a mechanism whereby the excitation of the complex RC1 directly allowed N–F cleavage to form the Selectfluor^®^ radical cation. The reaction of the C3 2° pentane radical with Selectfluor^®^ in an FAT was exergonic, with an energy barrier of 7.6 kcal⋅mol^−1^ relative to Int7, affording Int8 (Scheme 19). Since the Selectfluor^®^ radical cation is regenerated here upon FAT, the authors considered the candidacy of the Selectfluor^®^ radical cation as a chain carrier.

**Scheme 20:**
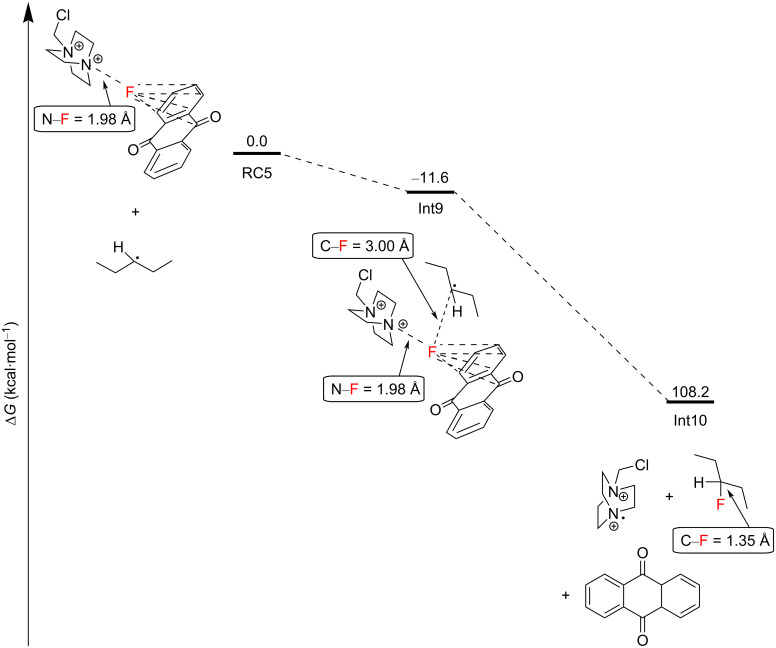
Barrierless fluorine atom transfer from Int1 to the C3 2° pentane radical to yield 3-fluoropentane, AQN and the Selectfluor^®^ *N-*radical cation.

However, the quantum yield of the reaction was found to be only Φ = 0.13, strongly evidencing against a radical chain mechanism. The complexation of Selectfluor^®^ with the PSCat in these reactions may inhibit its ability to participate in the propagation of a radical chain mechanism proposed for a complementary C(sp^3^)–H fluorination method [204]. Alternatively, FAT between the AQN–Selectfluor^®^ exciplex and the pentane C3 2° radical was found to be a highly exergonic process and could not be ruled out (Scheme 20).

#### 3.3 Directing-group-guided C(sp^3^)–H fluorination

**Scheme 21:**
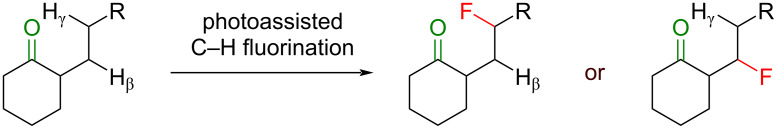
Ketone-directed C(sp^3^)–H fluorination.

**3.3.1 Ketones as directing groups:** Related to the curious near-exclusive C2 selectivity of 1-phenylbutan-1-one as reported by Tan and co-workers [198], Lectka and co-workers reported highly selective monofluorination of C(sp^3^)–H bonds that are proximal (β- or γ-) to a carbonyl group [153] (Scheme 21); such selectivity was previously difficult to achieve with PS TTET fluorinations.

**Scheme 22:**
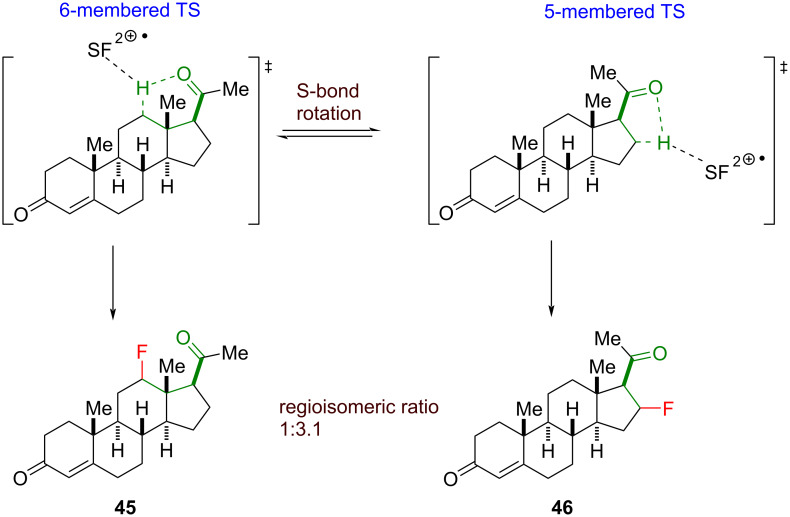
Ketone-directed fluorination through a 5- and a 6-membered transition state, respectively.

The ketones, acting as directing groups, served as an elegant method to override the typical polarity matching-governed C(sp^3^)–H fluorination [46,205]. The selectivity for β- or γ-fluorination could be attributed to an interaction of the breaking C(sp^3^)–H bond and the neighboring ketone, which is part of the proposed 5- and 6-membered transition state, respectively. When the 5- and the 6-membered transition state is possible, the free rotation of the σ-bond between the carbonyl group and the α-position allows the fluorination of the β- or γ-position. A good practical example of such a competitive fluorination is progesterone, which was fluorinated at both the C12 (see **45**) and the C16 (see **46**) position in a 1.0:3.1 ratio (Scheme 22). Ideally, to achieve the best selectivity, the β- and the γ-position must be distinctive based on their geometric constraints. We note that the nature of the transition states in this ketone-directed C(sp^3^)–H fluorination (and the subsequent carbonyl-directed C–H fluorinations) is not well-characterized, and we are tentative to draw firm conclusions on the regioselectivity of the C(sp^3^)–H fluorination at this stage.

**Scheme 23:**
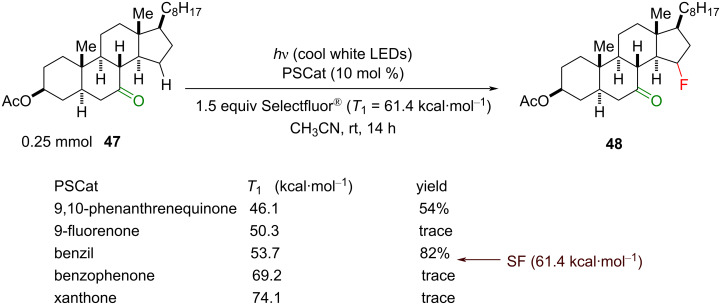
Effect of different PSCats on the photosensitized C(sp^3^)–H fluorination of **47**.

After screening several PSCats that absorb light >400 nm, such as benzophenone, 9-fluorenone, xanthone, 9,10-phenanthrenequinone and benzil, the highest yield (82%) was achieved with benzil (*T*_1_ = 53.7 kcal⋅mol^−1^, Scheme 23). Control reactions revealed that Selectfluor^®^ as the fluorinating agent gave the highest product yield, and light was essential for the reaction. To demonstrate the importance of the ketone directing group, ethylbenzene was employed under the same reaction conditions and no fluorinated product was observed, contrary to Chen’s previous report [201].

**Scheme 24:**
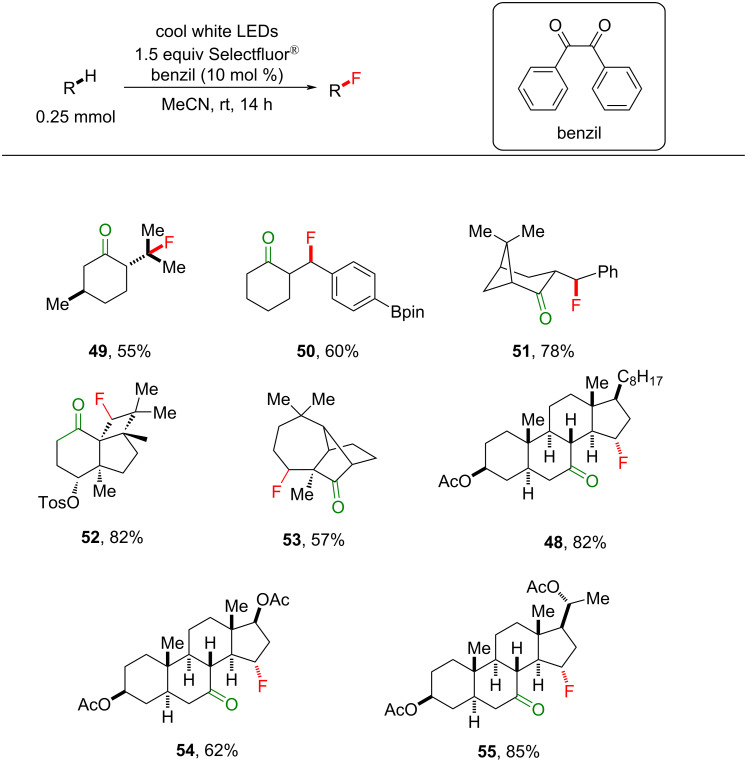
Substrate scope of benzil-photoassisted C(sp^3^)–H fluorinations.

The monofluorination of different classes of ketones, including 3° alkyl compounds or benzylic cyclohexanones, bicyclic ketones and steroidal ketones was achieved in moderate to excellent (43–85%) yield (**48**–**55**, Scheme 24), demonstrating the power of this method for LSF, especially given the biological activity and prevalence of steroids, such as cholesterol, progesterone and testosterone. Interestingly, under their conditions, 2-heptanone, 2-decanone and 2-dodecanone as the substrates gave in each case a distribution of fluorinated products, with a large preference for fluorination at the penultimate carbon (that would form a secondary radical), consistent with the polarity matching selectivity shown previously (Figure 10) [198,201].

Lectka and co-workers pointed out that the triplet energy of aliphatic ketones (79.4 kcal⋅mol^−1^ for acetone, Table 1) is too high to allow TTET from benzil and therefore triplet excited state intramolecular HAT of the substrate. They rationalized the distribution of the fluorinated products as a result of a radical chain reaction and suggested that linear ketones may promote cage escape of the Selectfluor^®^ radical cation to participate in this mechanism, in contrast to cyclic ketones. Under light-free conditions initiated by triethylborane (BEt_3_), they observed an identical monofluorination selectivity, which suggested the same intermediate (Selectfluor^®^ radical cation) in each case. Since these BEt_3_-initiated conditions are known to effect SET, they justified that the mechanism may occur through a PRC mechanism involving SET from the benzil triplet state to Selectfluor^®^. Given the lack of relationship between the *T*_1_ energy of the PSCats and the product yield in their optimization, this is a plausible suggestion. However, the lack of reactivity of certain PSCats due to them absorbing different visible (white LED) wavelengths cannot be ruled out.

**Scheme 25:**
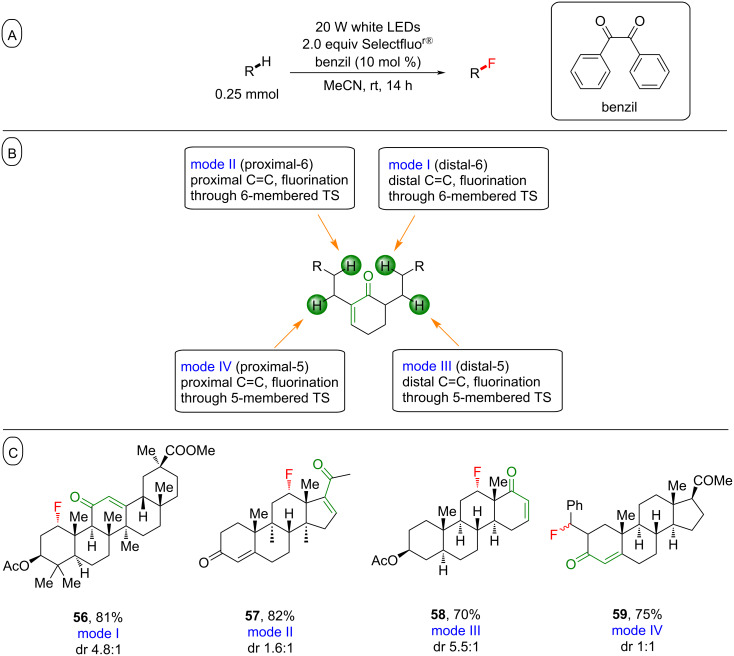
A) Benzil-photoassisted enone-directed C(sp^3^)–H fluorination. B) Classification of the reaction modes and transition states to rationalize the site-selectivity. C) C–H fluorination site-selectivity.

**3.3.2 Enones as directing groups:** Related to, and following their observations with cyclic ketone-containing substrates, Lectka and co-workers reported that enones also served as directing groups for PS TTET fluorinations [206]. C(sp^3^)–H bonds that were proximal (5 or 6 carbon atoms away) or distal (5 or 6 carbon atoms away) from the enone underwent C(sp^3^)–H fluorination to afford the products **56**–**59** in modest to very good yield (40–86%), with a high selectivity (Scheme 25). As before, a nonphotochemical reaction with Selectfluor^®^ and BEt_3_ provided a similar selectivity, corroborating the Selectfluor^®^ radical cation as an intermediate and suggesting that an SET mechanism may be possible.

**Scheme 26:**
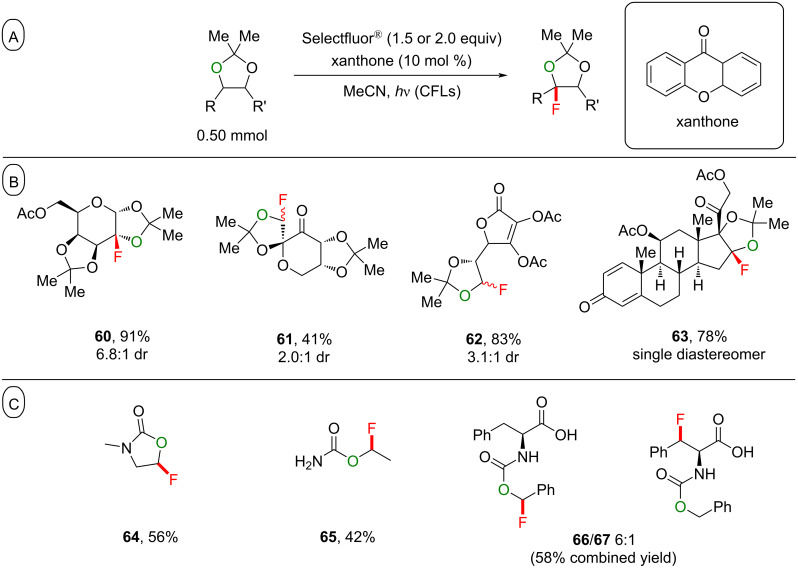
A) Xanthone-photoassisted ketal-directed C(sp^3^)–H fluorination. B) Substrate scope. C) C–H fluorination site-selectivity.

**3.3.3 Ketals as directing groups:** Very recently, Lectka, Dudding and co-workers reported the regioselective C(sp^3^)–H fluorination of acetonide ketals (Scheme 26A) [205]. C(sp^3^)–H bonds α to ketal oxygen atoms were fluorinated with surprisingly high selectivity to afford the products **60**–**63** in moderate to excellent yield (41–91%, Scheme 26B). The reaction conditions were also amenable to carbamates, affording the products **64**–**67** in moderate to good yield (42–58%). That the carbamate oxygen atom could direct the fluorination α to itself instead of α to the amide nitrogen atom provided an inverted, complementary selectivity as expected from the electrochemical oxidation (Scheme 26C).

**Scheme 27:**
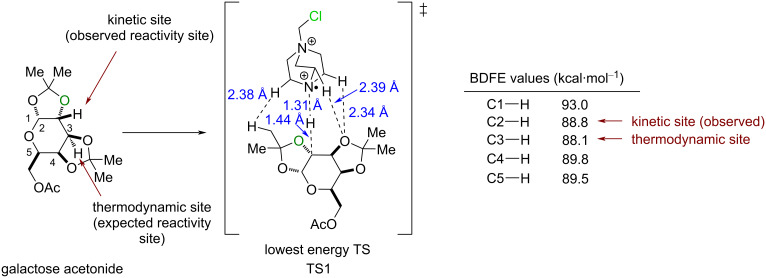
Rationale for the selective HAT at the C2 C–H bond of galactose acetonide.

When the ketals were not fused to a central ring, the least-hindered C(sp^3^)–H bond reacted, as in the products **61** and **62**. When the ketals were fused to a central ring, as in galactose acetonide, the C2 position underwent a selective C(sp^3^)–H fluorination. The authors demonstrated that the transition state for Selectfluor^®^ undergoing HAT at this position (see **TS1**) was more kinetically accessible, despite the bond dissociate free energies (BDFEs) suggesting C3 functionalization (Scheme 27).

#### 3.4 Directing-group-guided benzylic C–H fluorination

**Scheme 28:**
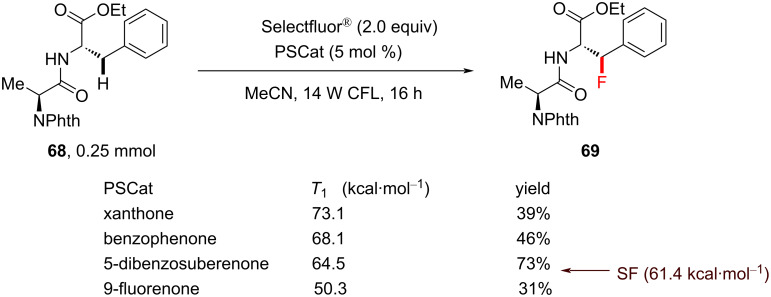
Photosensitized C(sp^3^)–H benzylic fluorination of a peptide using different PSCats.

In another report [207], Lectka and co-workers achieved selective monofluorination of the benzylic C–H bonds of phenylalanine- and tyrosine-like residues in peptides under visible-light photosensitization. After screening a variety of sensitizers, such as 1,2,4,5-tetracyanobenzene, AQN, 1,4-dicyanobenzene, 1-cyanonaphthalene, 9,10-phenanthrenequinone, xanthone, 2,7-dichloro-9-fluorenone, 9-fluorenone, benzophenone, 2-bromo-9-fluorenone and 2-chlorothioxanthone, 5-debenzosuberenone afforded the best yield of **69** (73%, Scheme 28).

**Scheme 29:**
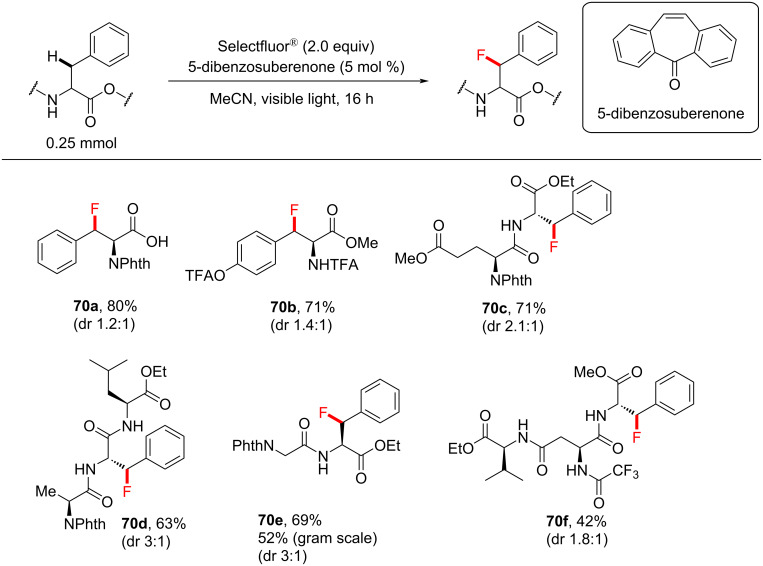
Peptide scope of 5-benzosuberenone-photoassisted C(sp^3^)–H fluorinations.

In the presence of protected basic, acidic and nonpolar side chains, benzylic fluorination was achieved with exclusive regioselectivity in peptides, with modest to moderate diastereomeric ratios of the fluorinated products (Scheme 29). With the optimum PSCat in hand, it was necessary to find a protecting group (PG) for the amine that would not undergo fluorination or oxidation when treated with Selectfluor^®^ [153]. The authors justified the use of protecting groups due to their extensive use in peptide synthesis. Of all the PGs tested, phthalimide (Phth)- and trifluoroacetate (TFA)-protected substrates underwent photosensitized C–H fluorination to give the highest yield of 80% and 71% of the respective products. Employing the optimum reaction conditions, Lectka and co-workers fluorinated phenylalanine-containing di- and tripeptides to give the products **70a**–**f** in modest to excellent yield (34–84%, Scheme 29). The carboxylic acid function could be free or protected as an ester without noticeable differences in the product yield.

A mechanism was not proposed in this study. The highest yield was obtained when the *T*_1_ energy of the PSCats and Selectfluor^®^ matched closely, suggesting that PS TTET may occur. However, a PRC mechanism involving SET cannot be ruled out, which the authors reported previously for benzylic fluorination [130]. In a later perspective [36], the authors speculated that the amide served as a directing group. Indeed, in situ-formed ammonium carbamates were recently identified to direct the HAT of a quinuclidinium radical cation [208].

#### 3.5 Scalability of C–H fluorinations

That the yields of gram-scale photosensitized C(sp^3^)–H fluorinations (Scheme 8 and Scheme 10) did not decrease indicates a fundamental advantage of PS TTET chemistry vs PRC; the low extinction coefficients of small-molecule PSCats in the near-UV to visible region (380–420 nm). In this respect, PS TTET photochemistry with near-UV behaves similar to UV photochemistry. On the other hand, photocatalysts typically used in PRC possess very high extinction coefficients, leading to a strong visible light absorption and reactions that suffer more on a scale-up due to the exponential relationship between the transmitted intensity of the light and the extinction coefficient, as described by the Beer–Lambert law (Equation 1).

[1] $$A=\log_{10}\cdot \frac{I_{0}}{I}=\varepsilon \cdot l\cdot c$$

Rearranging for *I* (Equation 2) and solving for a typical PSCat concentration (a reaction mixture of 0.1 M with 5 mol % PSCat; *c* = 5.0 mM) and the molar extinction coefficient for 9-fluorenone (ε = 270 M^−1^⋅cm^−1^ at 383 nm) [209–210] reveals a path length of *l* = 0.74 cm at which distance (from the surface) 90% of the light is absorbed. In comparison, a PRC reaction at the same concentration (*c* = 5.0 mM) involving the prototypical catalyst [Ru(bpy)_3_]Cl_2_ (ε = 11280 M^−1^⋅cm^−1^ at 452 nm) gives a path length of *l* = 17.7 mm at which distance (from the surface) 90% of the light is absorbed.

[2] $$l=\frac{\log_{10}\cdot \frac{I_{0}}{I}}{\varepsilon \cdot c}$$

**Scheme 30:**
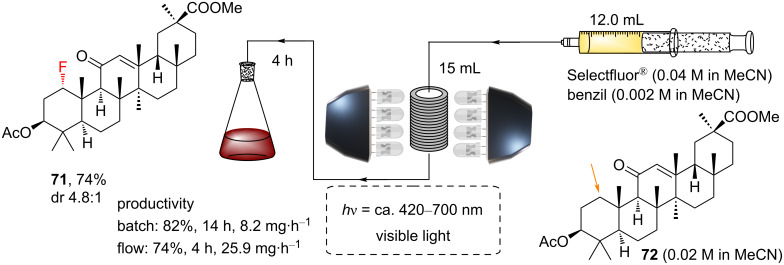
Continuous flow PS TTET monofluorination of **72**.

Flow chemistry provides an elegant means for scaling photochemical reactions [211–214], primarily due to the shorter path lengths for light transmission provided by flowing the reaction mixture through small-diameter (μm–mm) channels. Lectka and co-workers reported the processing of their benzil-photosensitized enone-directed C(sp^3^)–H fluorination in a custom-built flow reactor, comprised of a syringe pump, a coil of fluorinated ethylene propylene (FEP) tubing (7.5 m; inner diameter 1.6 mm; outer diameter 3.2 mm; representing a 15 mL internal volume) and 6 × 20 W 72-LED work light sources (white LEDs, Scheme 30). The reaction mixture (a 12 mL slug) was pumped through. After 4 h, the coil was flushed through with MeCN to collect all of the reaction mixture.

In the synthesis of **71**, the flow method gave, after 4 h, a similar (if slightly lower) yield vs the batch method after 14 h, reflecting a ≈ 3 times higher mass productivity. The benefit of flow photochemistry was not as obvious here compared to the batch due to the tolerance of the PS TTET batch reactions to scale-up on a laboratory scale (thanks to the low extinction coefficients as mentioned above). However, batch reactors larger than having centimeter-order diameters would begin to suffer issues, and the benefits of flow photochemistry are expected to manifest toward industrial-scale applications.

### 4 C–H fluorination of visible-light-active molecules via photosensitization auxiliaries (PSXs)

**Scheme 31:**
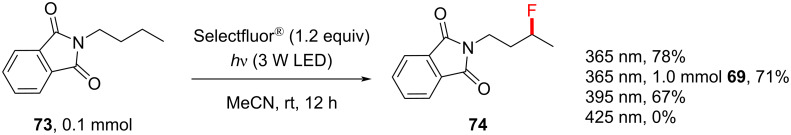
Photosensitized C–H fluorination of *N*-butylphthalimide as a PSX.

Egami, Hamashima and co-workers discovered the photoinduced fluorination of phthalimide derivatives without an exogenous PSCat (Scheme 31) [147]. Here, phthalimide serves as a PSX by absorbing 320–360 nm light, corresponding to its n–π* transition [215] and a triplet energy of *T*_1_ = 3.1 eV = 71.5 kcal⋅mol^−1^ [148]. The authors found that 365 nm LEDs and Selectfluor^®^ facilitated the direct C–H fluorination of *N-*butylphthalimide (**73**) in MeCN without an exogenous PSCat to give a 78% yield of the fluorinated product **74** (Scheme 31). While 395 nm was still effective, 425 nm and the absence of light gave no product.

**Scheme 32:**
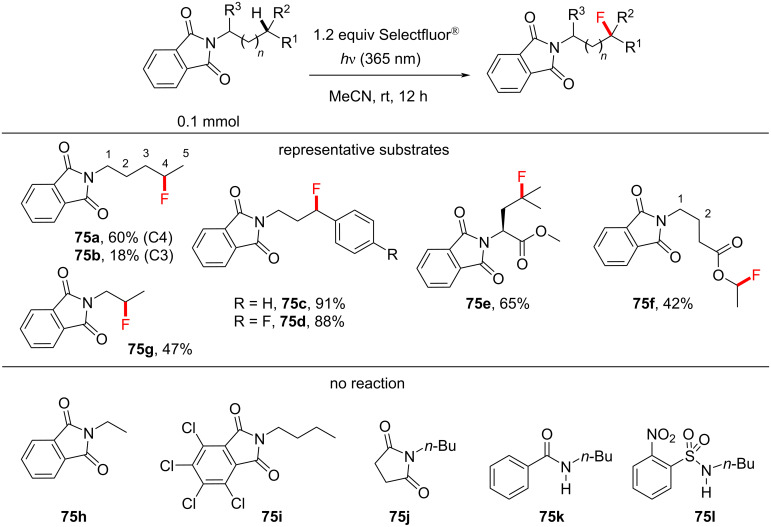
Substrate scope and limitations of the PSX C(sp^3^)–H monofluorination.

*N*-substituted phthalimides with alkyl chains underwent monofluorination at the most “hydridic” C(sp^3^)–H position, including 3° alkyl and benzylic positions, in moderate to near-quantitative (42–98%) yield. The functional group tolerance was not explored to a large extent; esters tolerated the reaction conditions (Scheme 32). The success and selectivity of the C–H fluorination of the *N*-alkylphthalimides depended strongly on the alkyl chain length. *N*-Pentylphthalimide gave the compounds **75a** and **75b** in a 3.2:1 ratio of the C4-/C3-monofluorinated product, *N*-propylphthalimide reacted to give a moderate yield of **75g** (47%) at the β-position, and *N*-ethylphthalimide (**75h**) gave no reaction (Scheme 32). The authors suggested that the C–H BDFE could explain the direction of the selectivity to the position furthest from the phthalimide, which formed the most stable radical, although we note that the selectivity in the fluorination of *N*-pentylphthalimide mirrored the selectivity of previous reports [198,201], which could be explained by polarity matching of the most “hydridic” C–H bond.

**Scheme 33:**
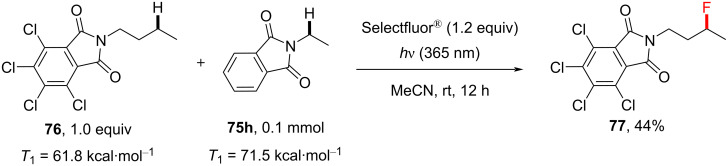
Substrate crossover monofluorination experiment.

One notable difference was the product **75f**, where fluorination occurred α to the ester oxygen atom, in contrast to the aforementioned PS TTET fluorination of the amyl benzoate **29**. One could speculate that an initial polarity matching-favored HAT at the C2 position might facilitate an intramolecular 1,5-HAT to generate a more stable radical for FAT. In order to examine the necessity of the phthalimide moiety in such reactions, the authors substituted it with nosyl, benzamide and succinimide groups, yet none of these molecules underwent a C(sp^3^)–H fluorination of the *n*-butyl chain. Although the 4,5,6,7-tetrachlorophthalimide **76** can easily be deprotected and employed in synthesis as a formal ammonia equivalent [147,216–218], the photosensitized C(sp^3^)–H fluorination of the substrate **76** was unsuccessful. However, when **76** was treated under the reaction conditions in the presence of *N*-ethylphthalimide, C–H fluorination of the former was observed (Scheme 33), clearly indicating that phthalimide is crucial for the fluorination reaction. Presumably, the triplet energy [172] of **76** (for 4,5,6,7-tetrachlorophthalimide: *T*_1_ = 61.8 kcal⋅mol^−1^) was too low to allow the successful reaction with Selectfluor^®^ (*T*_1_ = 2.6 eV, 61.4 kcal⋅mol^−1^). Although this energy is very similar to that of Selectflour^®^, it is possible that a certain amount of energy is lost during the reorganization/solvation of the excited state and complexation with Selectfluor^®^, such that **76** becomes an ineffective PSX.

**Scheme 34:**
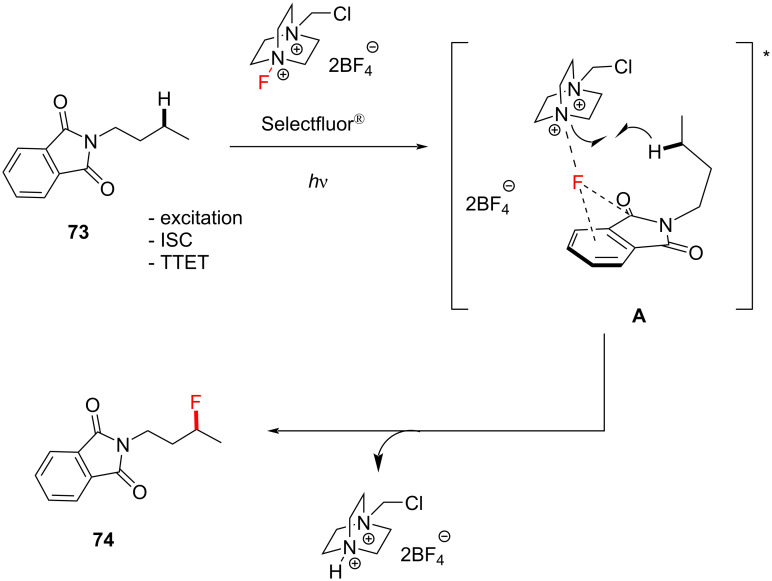
PS TTET mechanism proposed by Hamashima and co-workers.

Hamashima, Egami and co-workers proposed a PS TTET reaction mechanism consistent with that reported by Tan and co-workers [147,198] (Scheme 34), which leads to the exciplex **A**. Intramolecular HAT then occurs between the developing nitrogen radical cation of the Selectfluor^®^ moiety and the most hydridic C–H bond, which also benefits from the greatest thermodynamic stability of the generated radical. The authors then proposed that FAT occurs within the complex or between the formed radical and Selectfluor^®^ to afford the ﬂuorinated product. In contrast to previous methods involving PSCats, one fundamental advantage of this PSX approach is the ability to deconvolute the triplet energy matching of the PSCat and the fluorination source. One can simply use the same auxiliary to activate the substrate to fluorination and direct its selectivity before cleaving (for *N-*substituted phthalimides, by means of base hydrolysis or hydrazine [147]).

### 5 Trifluoromethylations involving photosensitization

**Scheme 35:**
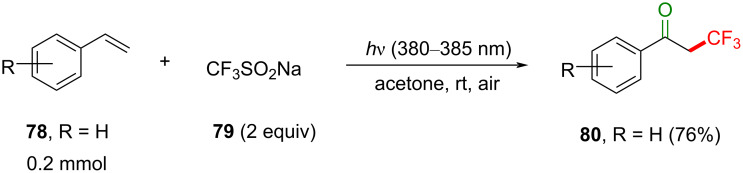
Photosensitized TFM of **78** to afford α-trifluoromethylated ketone **80**.

While most reported trifluoromethylation (TFM) methods involve PRC (SET) [219–224], limited examples of TFM involve photosensitization (energy transfer), and these will now be presented. A developing sub-class of PRC reactions involve the use of electron donor–acceptor (EDA) complexes [219,222], and these have been employed in TFMs. However, the reaction of the EDA complex in these methods is proposed to proceed via SET, and so these are beyond the scope of this review. Although the following example is not technically a C–H fluorination but rather an addition of a trifluoromethyl radical to an olefin, it includes a photosensitized energy transfer process. Li, Wang and co-workers achieved the TFM of simple styrenes to afford α-CF_3_-substituted ketones under mild conditions without using an external PSCat [225]. Their optimal reaction conditions employ CF_3_SO_2_Na (**79**, Langlois reagent) as a commercial and straightforward-to-handle CF_3_ source, air atmosphere and LED irradiation of 380–385 nm at rt (Scheme 35).

**Scheme 36:**
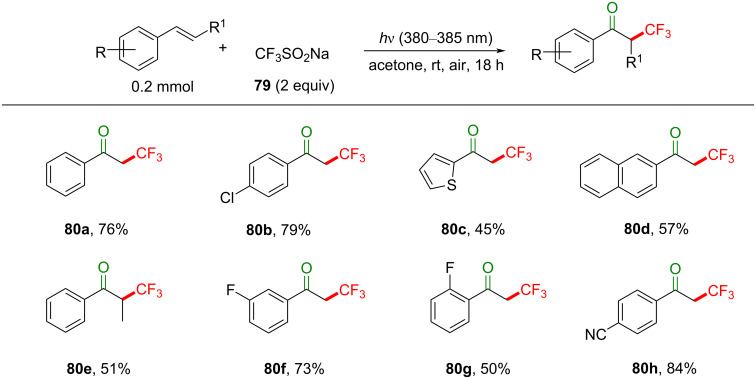
Substrate scope for photosensitized styrene TFM to give α-trifluoromethylated ketones.

Control reactions revealed that 380–385 nm light and acetone as a solvent are optimal for the reaction. Reactions in the dark or under an inert atmosphere did not proceed. Under the optimal conditions, a variety of styrenes substituted with EDG (such as Me, *t*-Bu, MeO) and EWG (such as Cl, Br, CF_3_, Ph, CN, CO_2_Me) on the aromatic ring were tolerated, giving the products **80a**–**h** in moderate to excellent product yield (45–84%, Scheme 36), with higher yields observed for electron-poor styrenes. Presumably due to steric effects, *meta*-substituted styrenes gave a higher product yield than *ortho*-substituted congeners. Nonstyrenic alkenes, such as cyclopentene, cyclooctene and benzyl vinyl ether gave no reaction.

**Scheme 37:**
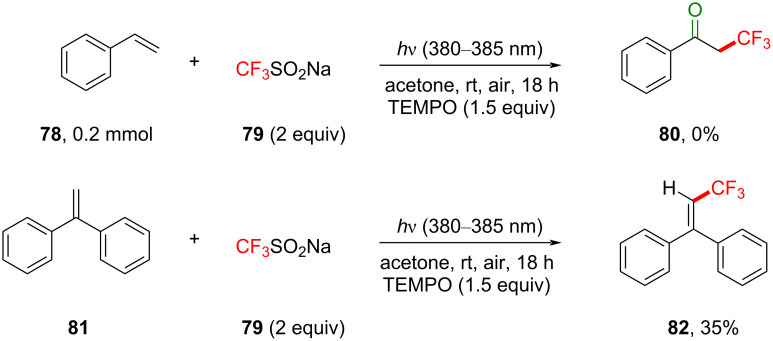
Control reactions for photosensitized TFM of styrenes.

Control reactions were chosen to provide mechanistic information on the TFM reaction (Scheme 37). In the presence of 1.5 equiv of (2,2,6,6-tetramethylpiperidin-1-yl)oxyl (TEMPO), the TFM reaction did not proceed, corroborating radical intermediates in the mechanism. Moreover, 1,1-diphenylethylene was subjected under the reaction conditions and reacted to afford the radical coupling product **82** (35%), implicating that CF_3_ radicals are generated (Scheme 37). We note that this control experiment constitutes a formal C(sp^2^)–H TFM.

**Scheme 38:**
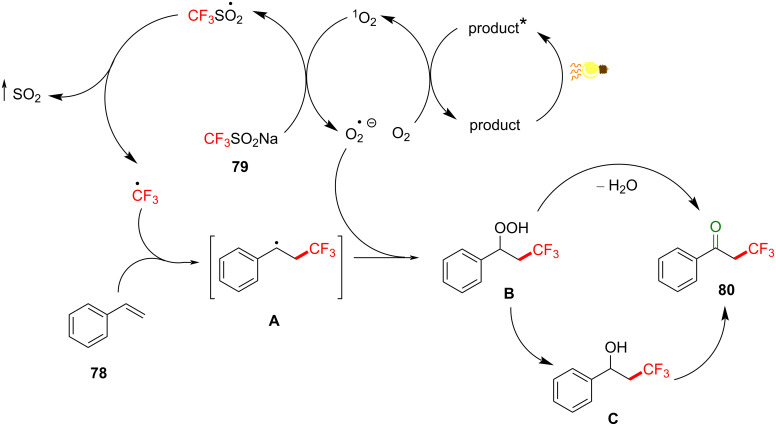
Reaction mechanism for photosensitized TFM of styrenes to afford α-trifluoromethylated ketones.

Electron paramagnetic resonance (EPR) in the presence of 5,5-dimethylpyrroline-*N-*oxide (DMPO) and light (380–385 nm) revealed a signal corresponding to the superoxide ion (O_2_^•−^), while the presence of 2,2,6,6-tetramethylpiperidine (TEMP) and light (380–385 nm) revealed a signal corresponding to singlet oxygen (^1^O_2_). Importantly, the fact that the products (α-trifluoromethylated ketones) can absorb light (380–385 nm) suggest their role in PS TTET in combination with O_2_, and thus the TFM reaction occurs by an autocatalytic manifold (Scheme 38). The mechanism starts with the excitation of the product by visible-light irradiation (380–385 nm, Scheme 38), which transfers its triplet energy to ground state triplet oxygen (^3^O_2_) to afford excited ^1^O_2_. This does not follow the previous definition of TTET and instead constitutes an example of a singlet–triplet energy transfer (STET) [226–227] or type II photosensitized oxygenation reaction [228].

Singlet oxygen reacts with **79** through SET to afford a superoxide ion (O_2_^•−^) and a CF_3_SO_2_^•^ radical, the latter of which decomposes to liberate SO_2_ gas and to release the CF_3_^•^ radical, which adds to the terminal styrene carbon atom to yield the intermediate **A**. The superoxide ion is then proposed to react with the intermediate **A** to yield the hydroperoxide intermediate **B**, which, after losing water, gives the product **80**. The authors proposed that the benzylic alcohol **C** might also serve as an intermediate en route to the product **80**. However, the initial generation of the product in the first place is unclear.

**Scheme 39:**
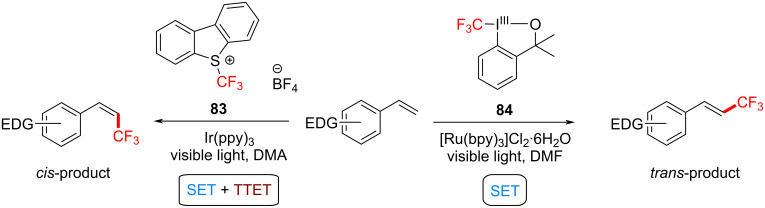
Reaction conditions for TFMs to yield the *cis*- and the *trans*-product, respectively.

Although the following example involves SET processes, it also includes a PS TTET process. A stereoinducing PS TTET trifluoromethylation was performed by Qing and co-workers [229]. They conducted direct and regioselectivity-controllable C(sp^2^)–H TFMs of electron-donating-group-substituted styrenes by employing either i) Togni’s reagent in the presence of catalytic [Ru(bpy)_3_]Cl_2_⋅6H_2_O and visible-light irradiation or ii) Umemoto’s reagent in the presence of catalytic Ir(ppy)_3_ and visible-light irradiation. The former proceeds under an SET mechanism to stereoselectively afford trifluoromethylated (*E*)-styrene, while the latter proceeds following both an SET and a TTET mechanism to afford a trifluoromethylated (*Z*)-styrene (Scheme 39).

**Scheme 40:**
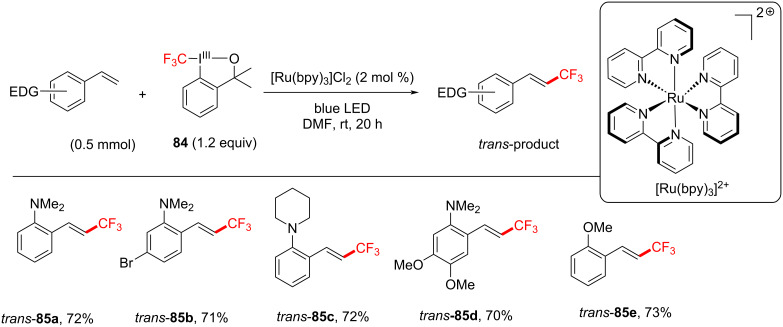
Substrate scope of trifluoromethylated (*E*)-styrenes.

**Scheme 41:**
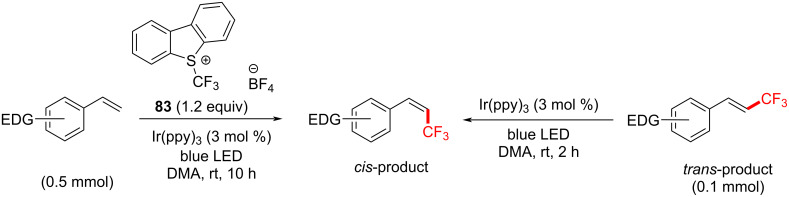
Strategies toward trifluoromethylated (*Z*)-styrenes.

**Scheme 42:**
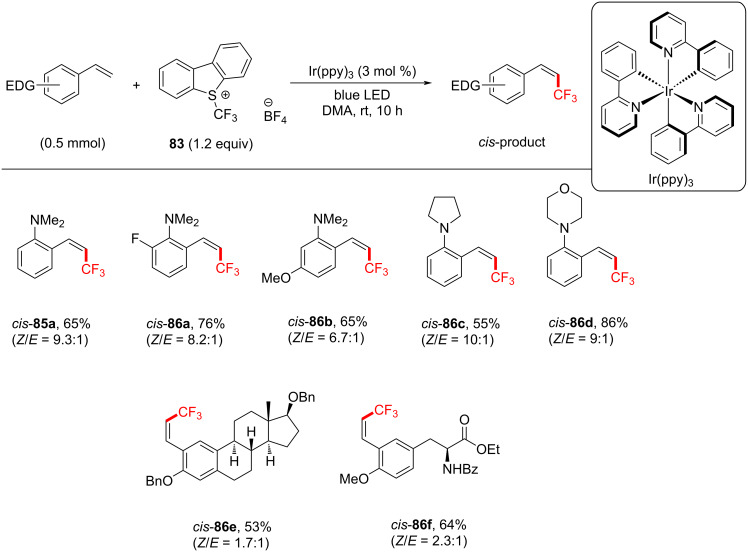
Substrate scope of trifluoromethylated (*Z*)-styrenes.

Notable byproducts were observed when styrene (**78**) itself was exposed to the reaction conditions. Attaching an EDG on the *ortho*-position suppressed the byproduct formation. Consequently, *N*,*N*-dimethyl-2-vinylaniline was subjected to TFM conditions and afforded *trans*-**85a** (72%) without any detectable byproduct after 10 h. The optimum set of conditions targeting *E*-isomers were: blue LEDs [230], room temperature and Togni’s reagent in DMF for 20 h (Scheme 40). Under these conditions, various EDG-substituted styrenes were tolerated in good to very good yield (50–78%). The synthesis of the *Z*-isomer could be performed in two ways (Scheme 41). Firstly, by reacting the isolated *E*-isomer with Ir(ppy)_3_ under blue LED irradiation in dimethylacetamide (DMA). Secondly (more elegantly), by reacting styrenes directly with Umemoto’s reagent, Ir(ppy)_3_ (3 mol %) as a catalyst under blue LED irradiation in DMA. Under the optimized conditions, in the latter case, various EDG-substituted styrenes, including steroid and amino acid-derived substrates were tolerated in good to very good yield (55–75%, Scheme 42), demonstrating the potential opportunities of this method in the LSF of bioactive molecules.

**Scheme 43:**
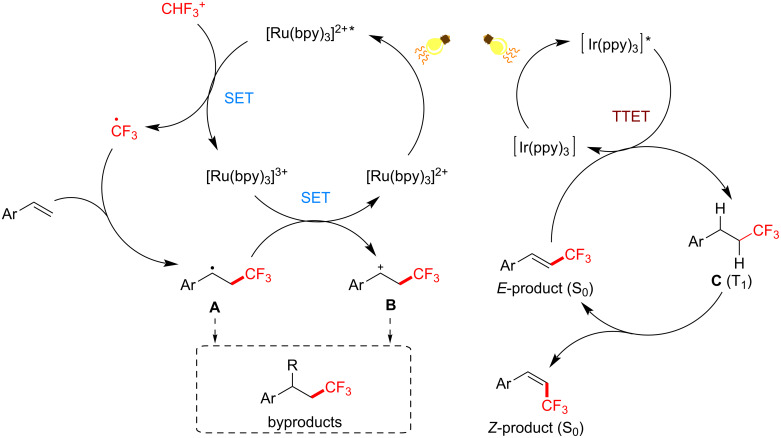
Reaction mechanism for photosensitized TFM of styrenes to afford *E*- or *Z*-products.

According to the mechanistic study of their first set of conditions, [Ru(bpy)_3_]^2+^ is excited by visible light to its excited state [Ru(bpy)_3_]^2+^* (*T*_1_ = 48.9 kcal⋅mol^−1^), which is oxidatively quenched by the electrophilic trifluoromethylating reagent (Scheme 43), affording [Ru(bpy)_3_]^2+^ and the trifluoromethyl radical. The latter radical reacts with the aromatic olefin to afford the stable, benzylic radical intermediate. SET oxidation of this radical by [Ru(bpy)_3_]^2+^ affords a β-CF_3_-substituted benzylic carbocation, which is deprotonated to furnish the (thermodynamically favored) *E*-isomer of the olefin. The authors rationalized that the EDG was required to increase the reactivity of intermediates and to suppress the formation of byproducts.

Previous studies on PS TTET involving transition metal complexes that promote olefinic *E*-to-*Z* isomerization [231] inspired Qing and co-workers to choose a PSCat with a higher triplet energy; Ir(ppy)_3_ (*T*_1_ = 55.6 kcal⋅mol^−1^) [232]. Here, Ir(ppy)_3_ facilitates the same oxidative quenching SET mechanism as [Ru(bpy)_3_]^2+^ to afford the *E*-isomer of the product. Thereafter, PS TTET between Ir(ppy)_3_ and product generates the triplet state of **C**, which is relieved of its π-bond and can freely rotate. Contrary to classical *cis*/*trans* isomerism of olefins under UV irradiation, where the thermodynamic preference for the *trans*-isomer is overridden by the higher extinction coefficient of the *trans*-isomer [233], herein, the styrene products do not absorb light at the wavelengths employed. The authors proposed that the driving force for the isomerism to the (*Z*)-olefin product may be due to a more efficient TTET between Ir(ppy)_3_* and the (*E*)-olefin. This is a reasonable argument since the triplet energy of the related (*cis*)*-*stilbene (*T*_1_ = 55.5 kcal⋅mol^−1^) is less accessible than that of the related (*trans*)-stilbene (*T*_1_ = 51.0 kcal⋅mol^−1^) [234]. Moreover, such triplet energies are likely out of the range of [Ru(bpy)_3_]^2+^ (*T*_1_ = 49.4 kcal⋅mol^−1^) but are likely within the range of Ir(ppy)_3_.

### 6 Future challenges

#### 6.1 Selectivity challenges

Despite the elegant efforts described herein, thus far, a key challenge that remains to be fully addressed in photosensitized C–H fluorinations (and direct C–H fluorinations in general) is the selectivity. Polarity matching-guided HAT is limited in scope not due to the PS TTET step but due to the inherent natural selectivity of the HAT step. Firstly, it provides a mixture of products despite the predictability of C–H fluorinations. Moreover, C–H bonds in proximity to functional groups such as alcohols and ketones are more reactive than the C–H bonds in alkyl chains elsewhere in the molecule. Finally, these methods are unable to functionalize the terminal positions of alkanes, which are oftentimes the desired positions of C–H fluorinations, due to the instability of the derived primary radicals. Directing groups for the HAT step, as elegantly identified by Lectka and co-workers [46,205–207], represent a complementary, if more valuable handle to direct selective C(sp^3^)–H fluorinations.

#### 6.2 Mechanistic understanding and precomplexation

More detailed studies are required to unearth the general reaction mechanisms in these PS TTET fluorinations. Comparing triplet energies is a useful guide but as can be seen comparing the reports on C(sp^3^)–H fluorinations of visible-light-inactive molecules using PSCats (Schemes 13, 21 and 26), this is not always an accurate predictor of the reactivity/success. The fact that the fluorination source (Selectfluor^®^) and light sources (generally 365–420 nm LEDs or CFL bulbs) are common themes but that different PSCats are optimal in different reactions suggests that the substrates themselves may play a role in the precomplexation with Selectfluor^®^ and the PSCat in order to module the UV–visible absorption or T_1_ energies [235–236]. Such behavior has been observed in other photochemical reactions [237–238].

#### 6.3 Applicability of the methods to industrial processes

Finally, the activity remains a general challenge in photochemical fluorinations. Industry demands that reactions proceed at a sufficient rate. While most reactions discussed herein require multiple hours reaction time, in radiolabeling applications, for example, the incorporation of ^18^F must be rapid, high-yielding and easily purifiable in order to synthesize, purify and characterize labeled molecules and get them into cells/organisms before the radioisotope decays away [97,239].

## Conclusion

In conclusion, we presented an account of PS TTET as an emerging and important method for the direct fluorination and trifluoromethylation of unactivated C–H bonds. This method provides a complementary selectivity to C–H fluorinations using classical methods or transition metal catalysis. It is also distinct from fluorination methods under PRC because it can be used to directly activate C(sp^3^)–H bonds instead of relying on the generation of radical intermediates by other methods (decarboxylation, addition of radicals to olefins). Moreover, it represents a highly practical and cost-effective method that can be scaled up without an erosion of the yield (at least to a multigram scale). The most promising facet of PS TTET fluorinations is the ability to directly functionalize C(sp^3^)–H bonds selectively, within complex molecules and under very mild conditions. Given the prevalence of C–F bonds in bioactive molecules, this method proves to be a powerful tool in LSF. Its scalability in batch thanks to the low extinction coefficient of the reaction mixtures renders this method rather amenable to production, with opportunities for further scale-up via flow photochemistry. This review should serve as a platform for the discovery of future PS TTET reactions and the mechanistic understanding thereof. We are particularly excited by the developments in directing-group-guided C(sp^3^)–H fluorinations of complex molecules and future investigations on the role of precomplexation and exciplex formation in the mechanisms of these reactions.

## List of Abbreviations

**Table 2:** Abbreviations

Abbreviation	Explanation

BDE	bond-dissociation enthalpy
BDFE	bond-dissociation free energy
CFL	compact fluorescence lamp
DCM	dichloromethane
EDA	electron donor acceptor (complex)
EPR	electron paramagnetic resonance
EWG	electron-withdrawing group
HAT	hydrogen atom abstraction
IC	internal conversion
ISC	intersystem crossing
LED	light-emitting diode
MeCN	acetonitrile
PC	photocatalyst
PHAT	photoexcited hydrogen atom transfer
PRC	photoredox catalysis
SET	single-electron transfer
SF	Selectfluor^®^
TFA	trifluoroacetic acid
TFM	trifluoromethylation
TM	transition metal
T.S.	transition state
TTET	triplet-triplet energy transfer
UV–vis	ultraviolet/visible
λ_max_	absorption maxima
τ	triplet-state lifetime
ns	nanosecond
μs	microsecond
